# Intraspecific Variation in Leaf Trait Responses to Experimental Warming in *Iris pumila* Populations from Contrasting Habitats

**DOI:** 10.3390/plants15213292

**Published:** 2026-10-25

**Authors:** Sanja Manitašević Jovanović, Katarina Hočevar, Veljko Vučković, Darka Šešlija Jovanović, Dušica Ćalić, Ana Vuleta

**Affiliations:** 1 Department of Evolutionary Biology, Institute for Biological Research “Siniša Stanković”—National Institute of the Republic of Serbia, University of Belgrade, 11108 Belgrade, Serbia; katarina.hocevar@ibiss.bg.ac.rs (K.H.); darka.seslija@ibiss.bg.ac.rs (D.Š.J.); ana.vuleta@ibiss.bg.ac.rs (A.V.); 2 Faculty of Biology, University of Belgrade, 11158 Belgrade, Serbia; b3014_2025@stud.bio.bg.ac.rs; 3 Department of Plant Physiology, Institute for Biological Research “Siniša Stanković”—National Institute of the Republic of Serbia, University of Belgrade, 11108 Belgrade, Serbia; calic@ibiss.bg.ac.rs

**Keywords:** *Iris pumila*, experimental warming, phenotypic plasticity, functional leaf traits, mechanistic leaf traits, phenotypic integration, population differentiation

## Abstract

Phenotypic responses to climate warming may differ among plant populations originating from contrasting habitats. To evaluate intraspecific variation in leaf trait responses to warming in *Iris pumila*, 40 genotypes from the Dune and Woods populations were exposed sequentially to ambient, +1 °C and +4 °C temperature regimes under controlled conditions. Functional leaf traits (specific leaf area, SLA, and leaf dry matter content, LDMC) and mechanistic leaf traits (specific leaf water content, SLWC; leaf thickness, LT, estimated from SLA and LDMC; stomatal density, SD; and the RGB-derived index I_1_, previously calibrated in *I. pumila* as inversely related to chlorophyll concentration) changed across the sequential temperature regimes. SLA, SLWC, LT and I_1_ increased, whereas LDMC and SD decreased. Dune and Woods genotypes differed in SLA, LDMC and I_1_, with higher SLA and I_1_ in Dune and higher LDMC in Woods. The phenotypic plasticity index (*PI*_V_) differed among leaf traits within each temperature contrast, with generally higher values for SD and I_1_ and lower values for SLA. Population differences in *PI*_V_ were evident for LDMC and SLWC from ambient to +1 °C (Dune > Woods) and for LDMC from +1 to +4 °C (Woods > Dune). Trait-correlation structure varied across the temperature sequence, although evidence for overall correlation-matrix similarity was not robust to changes in trait composition. No statistically detectable differences in the magnitude of phenotypic integration were found among temperature regimes or between populations. Overall, leaf responses in *I. pumila* revealed trait-specific changes and population-dependent patterns across the sequential temperature regimes, reflecting the complexity of intraspecific responses to warming.

## 1. Introduction

Anthropogenic climate warming is progressively altering the thermal conditions experienced by natural plant populations. Global surface temperature during 2011–2020 was approximately 1.1 °C above the pre-industrial level and further warming is expected over the coming decades under continued greenhouse-gas emissions [1]. Climate change is also altering temperature variability and seasonality, as well as the frequency and intensity of extreme climatic events [2]. Changes in the thermal environment can affect plant phenology, physiological performance and productivity by influencing plant metabolism, growth and development. The resulting plant responses depend on both the magnitude and temporal characteristics of temperature exposure [3]. Physiological adjustments to changes in growth temperature are commonly referred to as thermal acclimation [4]. Because plants are sessile, their capacity for *in situ* phenotypic adjustment is particularly important. Phenotypic plasticity—the ability of a genotype to express different phenotypes under different environmental conditions—can provide a rapid, within-generation response to environmental change [5,6,7], particularly when environmental conditions change faster than populations can respond through genetic evolution [6,8]. However, phenotypic plasticity is not inherently adaptive, as environmentally induced change may enhance or impair performance depending on the conditions [7,9,10]. Thus, the presence or magnitude of plasticity alone cannot be taken as evidence of adaptive capacity.

Leaf traits provide a valuable framework for examining plant responses to environmental change because they integrate variation in resource acquisition, tissue organization and physiological functioning [11,12]. Among the most commonly studied leaf traits, specific leaf area (SLA) and leaf dry matter content (LDMC) capture broad differences in resource-use and tissue-investment strategies, reflecting the trade-off between rapid resource acquisition and investment in more persistent tissues [13,14,15]. Because both traits are environmentally responsive, changes in their phenotypic expression can indicate shifts in leaf functional strategy under altered growth conditions.

However, the integrative nature of these functional traits may limit their ability to resolve more proximate components of plant responses to climate change. Brodribb [16] distinguished such functional traits from mechanistic traits that are more directly linked to specific physiological processes, although trait terminology and classification remain variable within trait-based ecology [17]. Kassout et al. [18] emphasized the complementary value of functional and mechanistic traits in plant-climate research, while more recent work has shown that mechanistically informative traits can improve the resolution of these relationships [19]. Accordingly, in the present study, SLA and LDMC were considered functional traits, whereas specific leaf water content (SLWC), estimated leaf thickness (LT), stomatal density (SD) and the RGB-derived chlorophyll-related index (I_1_) were treated as mechanistic traits representing complementary hydration–structural, stomatal–anatomical and chlorophyll-related components of the leaf phenotype.

Beyond their individual responses, functional and mechanistic traits may covary because they are components of the same phenotype and share functional, structural or developmental relationships [15]. Recent work has therefore emphasized that plant responses should be considered in terms of coordinated trait combinations rather than isolated traits [20]. Phenotypic integration, defined as covariation among functionally related traits within an organism [21], provides a framework for examining such associations and can be characterized by both the magnitude of covariation and the pattern of relationships among traits [22]. These relationships are not necessarily fixed, as environmental conditions can alter both the magnitude and pattern of trait correlations [21,23,24]. A recent meta-analysis further showed that phenotypic integration is associated with coordinated plastic responses among traits under environmental change [25]. Warming may therefore affect not only individual trait values and plasticity, but also the patterns of association among traits. Examining trait integration across temperature conditions can reveal whether these relationships are maintained or altered under warming.

The expression and coordination of leaf traits can vary among populations within species [26] and populations originating from contrasting environments may differ in mean trait expression and phenotypic plasticity [2,27,28]. Such intraspecific variation is relevant to responses to climate warming because species-level patterns may obscure population-level differences. The two natural populations of *Iris pumila* examined here originate from contrasting habitats in the Deliblato Sands: an open dune and a shaded woodland habitat, which differ markedly in irradiance and other abiotic conditions, including temperature regimes [26,29,30]. Differences expressed under common growth-room conditions may therefore reflect persistent differentiation between these populations in trait expression and temperature responsiveness.

Our previous experimental work showed that a 1 °C increase in growth temperature was sufficient to induce plastic changes in both functional and mechanistic leaf traits in *I. pumila* [31]. However, that study included a single source population and two temperature conditions, leaving unclear whether responses to moderate warming persist or change as temperature increases further and whether their magnitude and coordination differ between populations from contrasting habitats. In the present study, we examined leaf trait responses in clonal genotypes of *I. pumila* originating from Dune and Woods populations across a sequential increase in growth temperature from ambient to +1 °C and +4 °C. These increments represent lower and higher levels within the range of warming projected for this century [1]. Accordingly, we expected (i) leaf trait means and the magnitude of phenotypic plasticity to vary across the sequential temperature regimes; (ii) the two populations to differ in overall leaf trait levels and response patterns across these conditions; and (iii) the pattern and magnitude of leaf trait integration to vary across the temperature sequence and between populations. Together, these analyses were intended to provide a more comprehensive understanding of leaf responses across a sequential increase in growth temperature by considering phenotypic plasticity, population origin and trait integration jointly.

## 2. Results

### 2.1. Functional and Mechanistic Leaf Trait Responses Across the Temperature Sequence

Across the sequential increase in growth temperature, both functional and mechanistic leaf traits showed similar directional changes across populations (Table 1). SLA, SLWC, estimated LT and I_1_ generally increased across the temperature sequence, whereas LDMC and SD showed an overall decline. Mean SLA and I_1_ values were consistently higher in Dune genotypes across all temperature regimes, whereas LDMC was always higher in Woods genotypes. SLWC, LT and SD were generally higher in Woods; however, at +1 °C, SLWC and LT were higher in Dune, while SD was similar between populations.

The magnitude of trait responses differed across temperature regimes and between populations. Regarding functional traits, SLA increased gradually across the temperature sequence, reaching its highest values at +4 °C in both populations, whereas LDMC consistently decreased. Relative to ambient conditions, SLA increased by approximately 9% in both Dune and Woods at +4 °C, while LDMC decreased by 15% and 16%, respectively. Mechanistic traits showed more heterogeneous response patterns. SLWC exhibited a stronger early increase in Dune genotypes, reaching 15% at +1 °C and remaining elevated at +4 °C, whereas in Woods genotypes, it increased more progressively, from 6% at +1 °C to 15% at +4 °C. A similar population-specific pattern was observed for LT, with a stronger increase at +1 °C in Dune genotypes and a progressive increase across the temperature sequence in Woods (3% at +1 °C and 10% at +4 °C). SD showed a transient increase at +1 °C only in Dune, followed by a decline at +4 °C, while in Woods, it decreased more consistently across the temperature sequence (1% at +1 °C and 9% at +4 °C). I_1_ increased in both populations, reaching 14% in Dune and 13% in Woods at +4 °C.

These descriptive patterns were then formally evaluated using repeated-measures ANOVA, which revealed significant differences among the sequentially applied temperature regimes for all analyzed functional and mechanistic leaf traits (Table 2). SLA, LDMC, SLWC, LT, SD and I_1_ changed across the temperature sequence from ambient conditions to +1 °C and +4 °C. The population effect was significant for SLA (ηp^2^ = 0.385), LDMC (ηp^2^ = 0.255) and I_1_ (ηp^2^ = 0.313), showing that the mean response profiles of Dune and Woods genotypes differed in their overall levels for these traits. In contrast, no significant population differences were detected for SLWC, LT and SD when averaged across temperature regimes. The temperature × population interaction was significant for SLWC and LT, indicating that the hypothesis of parallel response profiles was rejected for these two mechanistic traits. Therefore, the trait patterns of SLWC and LT across the temperature sequence were examined further at the population level. In contrast, SLA, LDMC, SD and I_1_ showed broadly parallel response profiles between populations, with no significant temperature × population interactions detected.

Profile contrasts were then used to further examine changes across the temperature sequence in traits with broadly parallel population response profiles (Table S1). Among these traits, SLA, LDMC and I_1_ changed significantly already between ambient temperature and +1 °C, whereas no statistically detectable change in SD was observed during this initial warming step. Additional changes between +1 °C and +4 °C were detected for SLA, LDMC and SD, while I_1_ showed no significant further change between the two elevated temperature regimes. The direct comparison between ambient temperature and +4 °C confirmed significant overall differences for all traits with parallel response profiles. A significant population × contrast effect was detected only for LDMC between +1 °C and +4 °C, whereas no significant population × contrast effects were observed for the ambient vs. +1 °C or ambient vs. +4 °C comparisons.

For SLWC and LT, where the temperature × population interaction was significant, simple-effect comparisons were performed to clarify the population-specific patterns across temperature regimes (Table S2). These analyses showed that the non-parallel response of SLWC resulted from divergent dynamics after the initial warming step: SLWC increased from ambient temperature to +1 °C in both populations, but a further increase from +1 °C to +4 °C occurred only in Woods genotypes. Accordingly, the overall difference between ambient temperature and +4 °C was significant in both populations (Table S2A). Population differences within individual temperature regimes were significant only at +1 °C, where Dune genotypes exhibited higher SLWC than Woods genotypes (Table S2B). For LT, simple-effect comparisons revealed population-specific patterns across the successive temperature contrasts. In Dune, LT increased significantly from ambient temperature to +1 °C and showed no statistically detectable further change between +1 °C and +4 °C, whereas in Woods, LT increased significantly only between the two elevated temperature regimes. In both populations, the overall difference between ambient temperature and +4 °C was significant. Population differences within individual temperature regimes were significant only at +4 °C, where LT was higher in Woods than in Dune genotypes.

### 2.2. Phenotypic Plasticity of Functional and Mechanistic Leaf Traits Across Sequential Temperature Regimes

**Figure 1:**
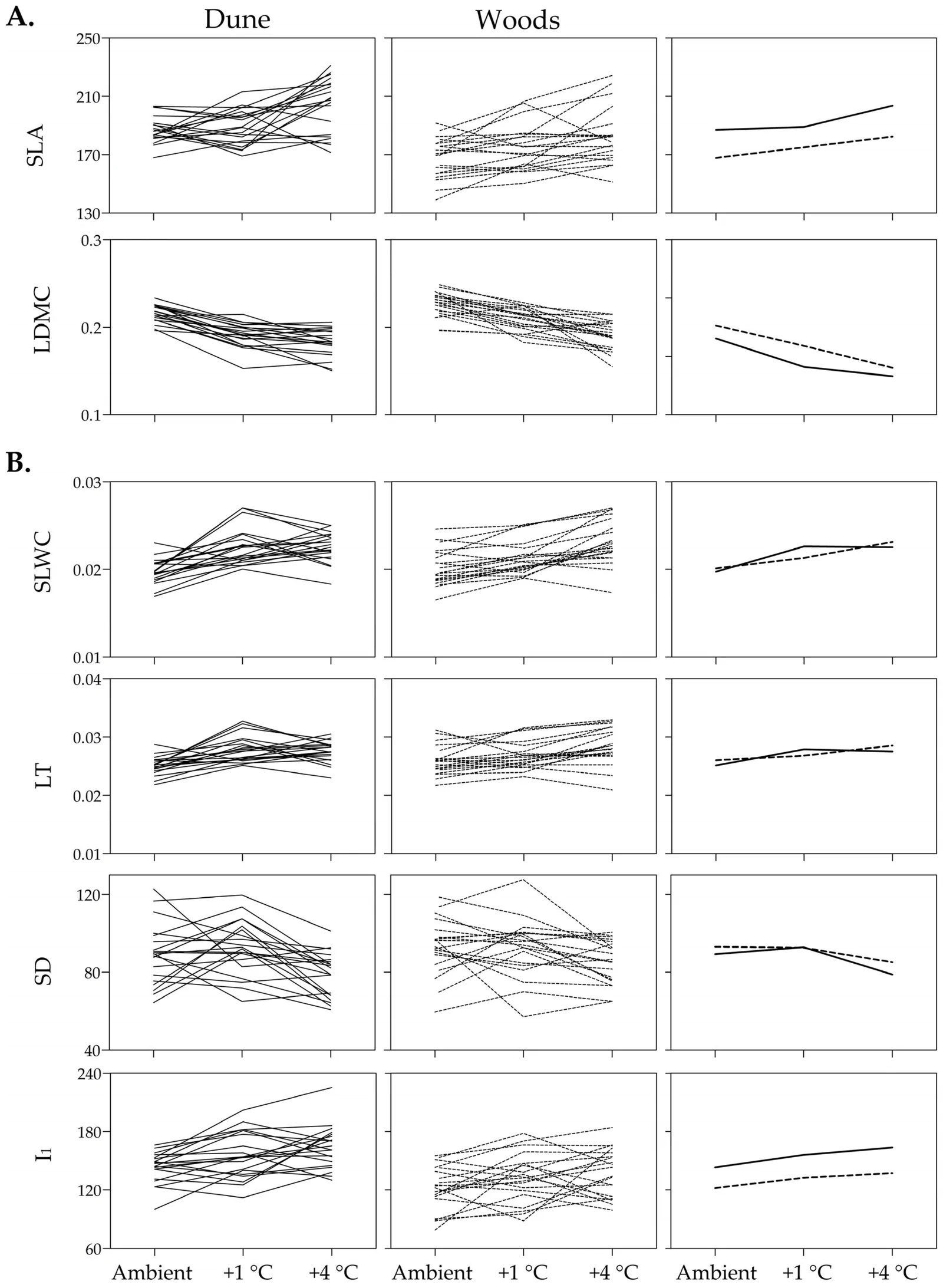
Reaction norms for the functional (**A**) and mechanistic (**B**) leaf traits in *I. pumila* genotypes originating from the Dune and the Woods populations, examined across sequential temperature regimes (ambient, +1 °C and +4 °C) in an environmentally controlled growth room. Mean trait values are shown in the right panels (Dune—solid lines; Woods—dashed lines). For trait acronyms and measurement units, see Table 1.

Reaction norms for the functional and mechanistic leaf traits of *I. pumila* genotypes originating from the Dune and Woods populations across sequential temperature regimes (ambient, +1 °C and +4 °C), together with the corresponding mean reaction norms, are presented in Figure 1. The relatively steep slopes of the mean reaction norms indicate phenotypic adjustment of *I. pumila* functional and mechanistic leaf traits across the temperature regimes. The observed reaction norm patterns differed among traits and, for selected traits, between populations. The reaction norms for SLA, SLWC, LT and I_1_ generally exhibited an upward trend across the temperature sequence, whereas that for LDMC showed a downward trend in both Dune and Woods genotypes (Figure 1). In contrast, SD displayed more complex response patterns, characterized by an initial increase at +1 °C followed by a decline at +4 °C, particularly in the Dune genotypes.

The reaction norms of individual genotypes displayed moderate variation within both populations, with occasional crossings observed for several traits, indicating shifts in genotype ranking under different temperature regimes. Divergence patterns were most evident for SLA and I_1_ at +1 °C and +4 °C, particularly in the Woods population, where genotypic reaction norms became more widely separated. In contrast, LDMC reaction norms tended to converge toward similar values across temperature regimes, whereas SD displayed more variable and less coordinated response patterns in both populations. SLWC and LT showed heterogeneous response patterns, with individual reaction norms remaining relatively dispersed across temperature regimes and without a clear tendency toward either convergence or divergence (Figure 1).

Variation among *I. pumila* genotypes, quantified by the coefficient of variation (CV%), differed among traits, populations and temperature regimes (Table S3). Functional traits generally exhibited lower variability than mechanistic traits, with LDMC showing consistently low CV% values across temperature regimes (4.6–8.7%), whereas I_1_ and SD were the most variable traits. The highest CV% values were observed for SD in Dune genotypes and I_1_ in Woods genotypes, reaching 17.4% and 19.1%, respectively. Changes in CV% across the temperature sequence differed between populations. Functional traits showed a similar overall trend in both populations, with variability increasing across the temperature sequence, although this increase was more pronounced in Dune genotypes. Mechanistic traits showed different patterns of variation between the two populations: in Dune genotypes, SLWC, LT and I_1_ reached their highest variation at +1 °C before declining at +4 °C, whereas SD decreased progressively from ambient conditions to +4 °C.

In Woods genotypes, SD and I_1_ also reached their highest variability at +1 °C, while SLWC and LT decreased slightly at +1 °C and then increased again under the highest temperature regime (Table S3). After Holm adjustment, paired Pitman–Morgan tests showed limited evidence of changes in phenotypic variance across temperature regimes within populations. In Dune genotypes, SLA variance differed significantly between ambient and +4 °C (*t* = −3.579, *p* = 0.006), while I_1_ showed a marginal difference between ambient and +1 °C (*t* = −2.373, *p* = 0.087). No other significant within-population differences in variance were detected, and none were significant in Woods genotypes (Table S4A). Between populations, two-sided *F*-tests detected no significant differences in variance at any temperature regime; only LT at +4 °C showed a marginal difference between Dune and Woods genotypes (*F* = 0.328, *p* = 0.057; Table S4B).

**Figure 2:**
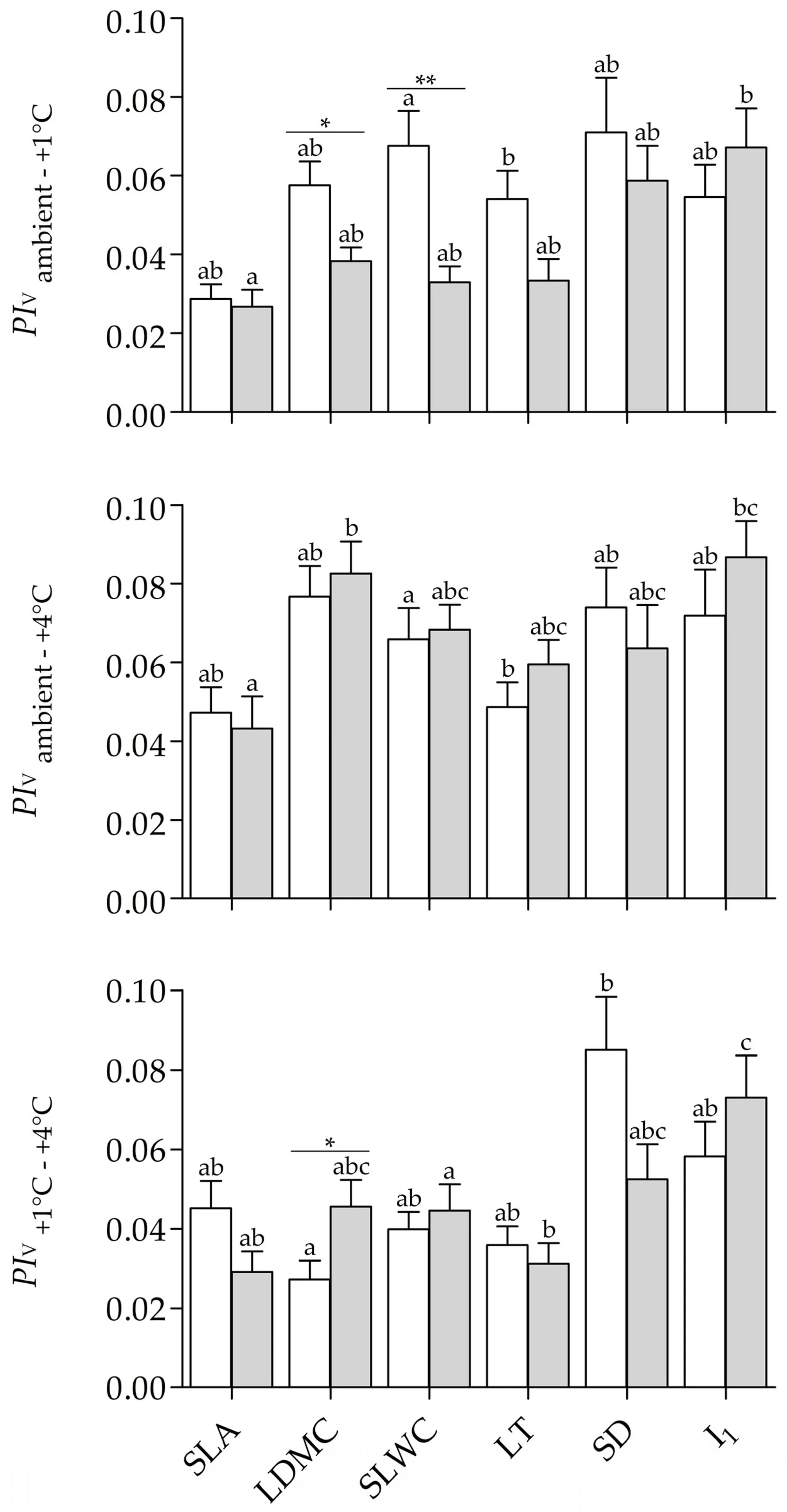
The phenotypic plasticity indices (*PI*_V_, mean ± SE) for the functional (SLA, LDMC) and mechanistic (SLWC, LT, SD, RGB index I_1_) leaf traits of *I. pumila* genotypes originating from the Dune (white bars) and Woods (grey bars) populations, calculated for the three temperature contrasts (ambient vs. +1 °C, ambient vs. +4 °C and +1 °C vs. +4 °C). Different lowercase letters indicate significant differences among traits within the same population and temperature contrast (Friedman ANOVA followed by Wilcoxon signed-rank tests with Holm-adjusted *p*-values, *p* < 0.05). Asterisks indicate significant differences between populations for the same trait within a given temperature contrast (Mann–Whitney tests with BH-adjusted *p*-values, * *p* < 0.05, ** *p* < 0.01).

To quantify the relative magnitude of phenotypic change between temperature regimes, plasticity indices (*PI*_V_) were calculated for three contrasts: ambient vs. +1 °C, ambient vs. +4 °C and +1 °C vs. +4 °C (Figure 2). Mean *PI*_V_ values ranged from 0.027 to 0.087 across leaf traits, populations and temperature contrasts. In Dune genotypes, SD showed the highest mean *PI*_V_ values under the ambient vs. +1 °C and +1 °C vs. +4 °C, whereas LDMC showed the highest mean *PI*_V_ value under the ambient vs. +4 °C contrast. In Woods genotypes, I_1_ showed the highest mean *PI*_V_ values in all three temperature contrasts.

The results of Friedman ANOVA, conducted separately for each population and temperature contrast, showed significant differences in *PI*_V_ values among leaf traits in both populations and across all temperature contrasts. In Dune genotypes, *PI*_V_ values differed significantly under the ambient vs. +1 °C (χ^2^(5) = 12.30, *p* = 0.03), the ambient vs. +4 °C (χ^2^(5) = 20.23, *p* = 0.001) and the +1 °C vs. +4 °C (χ^2^(5) = 16.74, *p* = 0.005) contrasts. Similarly, in Woods genotypes, significant variation among traits was observed under the ambient vs. +1 °C (χ^2^(5) = 24.08, *p* < 0.001), the ambient vs. +4 °C (χ^2^(5) = 17.85, *p* = 0.003) and the +1 °C vs. +4 °C (χ^2^(5) = 29.14, *p* < 0.001) contrasts.

Although the Friedman tests were significant in all population–contrast combinations, post hoc Wilcoxon signed-rank tests, with Holm adjustment applied separately within each population × temperature-contrast family of 15 pairwise comparisons, showed that significant differences were restricted to a limited number of trait pairs (Figure 2). In Dune genotypes, SLWC had significantly higher *PI*_V_ values than LT under both the ambient vs. +1 °C and the ambient vs. +4 °C contrasts (*p* = 0.007 and *p* = 0.004, respectively), whereas SD had higher *PI*_V_ values than LDMC under the +1 °C vs. +4 °C contrast (*p* = 0.005). In Woods genotypes, I_1_ had higher *PI*_V_ values than SLA under the ambient vs. +1 °C contrast (*p* = 0.047). Under the ambient vs. +4 °C contrast, SLA had lower *PI*_V_ values than both LDMC and I_1_ (*p* = 0.005 and *p* = 0.038, respectively), while under the +1 °C vs. +4 °C contrast, I_1_ had higher *PI*_V_ values than SLA, SLWC and LT (*p* = 0.013, *p* = 0.044 and *p* = 0.005, respectively), and SLWC had higher *PI*_V_ values than LT (*p* = 0.040).

Population comparisons revealed significant differences in *PI*_V_ values between Dune and Woods genotypes for only a limited number of traits and temperature contrasts. Mann–Whitney U tests with Benjamini–Hochberg (BH)-adjusted *p*-values revealed significant population differences for LDMC under the ambient vs. +1 °C (W = 296, *p* = 0.014) and +1 °C vs. +4 °C contrasts (W = 102, *p* = 0.014) and for SLWC under the ambient vs. +1 °C contrast (W = 315, *p* = 0.006). Under the ambient vs. +1 °C contrast, Dune genotypes exhibited higher mean *PI*_V_ values than Woods genotypes for both LDMC (*PI*_V_ = 0.058 vs. 0.038) and SLWC (*PI*_V_ = 0.068 vs. 0.033). Conversely, under the +1 °C vs. +4 °C contrast, the mean *PI*_V_ value for LDMC was higher in Woods than in Dune genotypes (*PI*_V_ = 0.046 vs. 0.027).

No significant population differences were detected for *PI*_V_ SLA, *PI*_V_ LT, *PI*_V_ SD, or *PI*_V_ I_1_ after correction for multiple comparisons. Differences in *PI*_V_ among temperature contrasts were then tested separately for each trait within each population. In Dune genotypes, Friedman ANOVA showed significant differences in *PI*_V_ only for LDMC (χ^2^(2) = 21.70, *p* < 0.001). Post hoc Wilcoxon signed-rank tests, with Holm adjustment applied to the three pairwise comparisons, showed significant differences between all three temperature contrasts: ambient vs. +1 °C and ambient vs. +4 °C (*p* = 0.015), ambient vs. +1 °C and +1 °C vs. +4 °C (*p* = 0.008) as well as ambient vs. +4 °C and +1 °C vs. +4 °C (*p* < 0.001).

In Woods genotypes, significant differences among temperature contrasts were detected for LDMC (χ^2^(2) = 19.20, *p* < 0.001), SLWC (χ^2^(2) = 13.23, *p* = 0.001) and LT (χ^2^(2) = 11.87, *p* = 0.003), whereas SLA showed only a marginal tendency (χ^2^(2) = 5.69, *p* = 0.058). For LDMC, SLWC and LT, post hoc comparisons showed that the ambient vs. +4 °C contrast differed from both the ambient vs. +1 °C and the +1 °C vs. +4 °C contrasts, whereas no statistically detectable difference was found between the latter two contrasts. These results indicate that differences in the magnitude of phenotypic change among temperature contrasts varied among traits and between populations.

A nonparametric longitudinal analysis was additionally performed to evaluate the effects of population, temperature contrast and their interaction on *PI*_V_ (Table S7). Because population differences in *PI*_V_ among temperature contrasts were examined in detail in the preceding analyses, the nonparametric longitudinal analysis is reported here primarily to evaluate the Population × Temperature contrast interaction. Significant interactions were detected for *PI*_V_ LDMC (ATS = 9.09, df = 1.40, *p* < 0.001), *PI*_V_ SLWC (ATS = 6.40, df = 1.91, *p* = 0.002) and *PI*_V_ LT (ATS = 4.16, df = 1.85, *p* = 0.018), indicating that the pattern of *PI*_V_ across temperature contrasts differed between Dune and Woods genotypes for these traits. In contrast, no significant interaction was found for *PI*_V_ SLA, *PI*_V_ SD or *PI*_V_ I_1_.

### 2.3. Patterns of Trait Correlation and Integration Across Temperature Regimes and Populations

**Figure 3:**
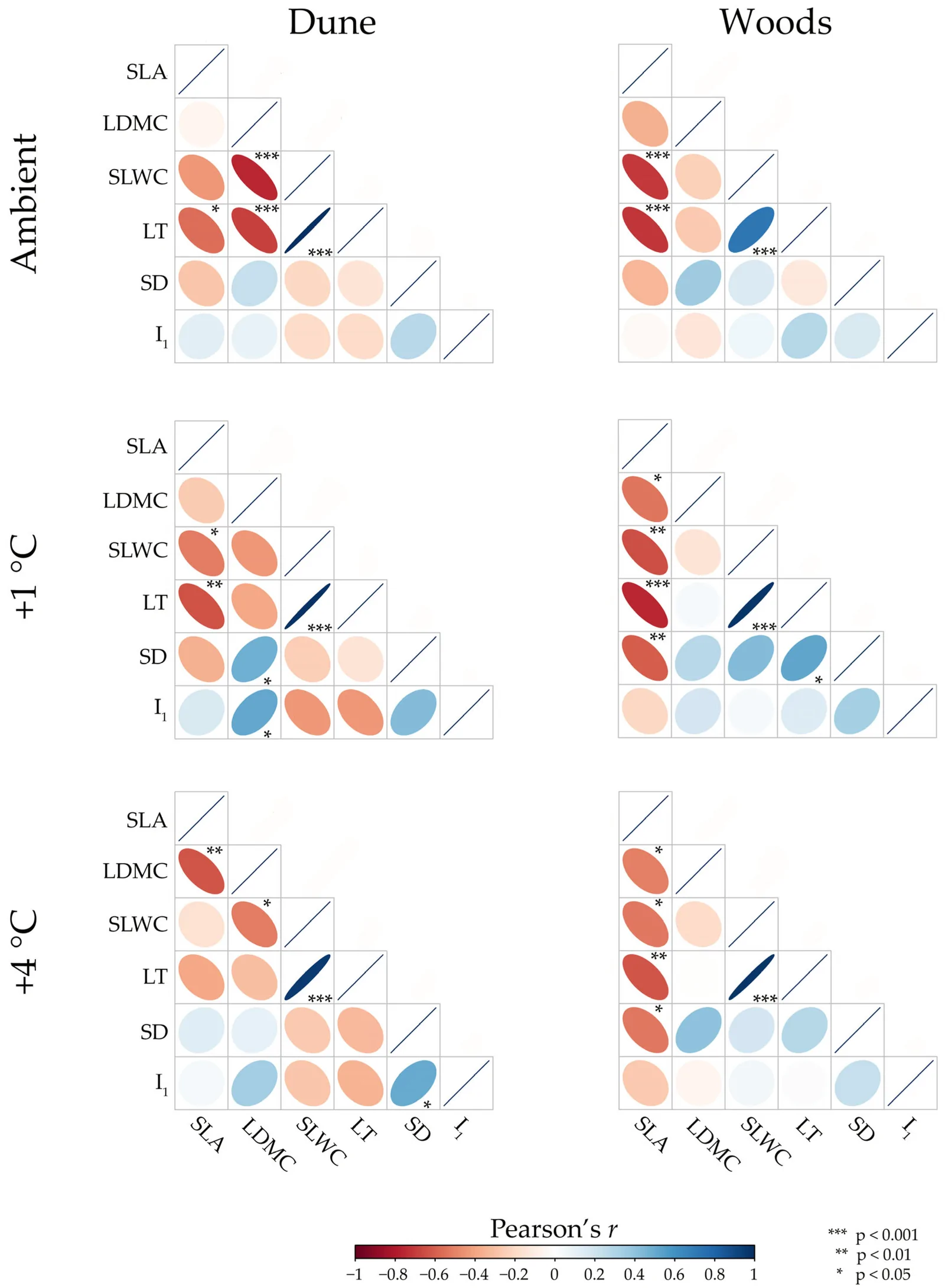
Heat maps of the correlations between the functional (SLA and LDMC) and mechanistic (SLWC, LT, SD, RGB-derived index I_1_) leaf traits in *I. pumila* genotypes originating from the Dune and the Woods populations across sequential temperature regimes (ambient, +1 °C and +4 °C). The color gradient and the ellipse eccentricity represent the strength and direction of the correlations, with blue denoting stronger positive associations and red denoting stronger negative ones. Correlation coefficients are classified as weak (|r| < 0.4), moderate (0.4 ≤ |r| ≤ 0.8), or strong (|r| > 0.8). Significance levels are indicated by asterisks: * *p* < 0.05, ** *p* < 0.01, *** *p* < 0.001. For trait acronyms and measurement units, see Table 1.

The associations among functional and mechanistic leaf traits were examined separately for Dune and Woods genotypes under each temperature regime (Figure 3). Significant correlations were present in all population-by-temperature combinations, but their number, sign and distribution varied across temperature regimes and populations. Correlations involving traits that share underlying measurements or derived relationships are reported descriptively but are not treated as independent evidence of biological trait coordination. All significant correlations were at least moderate in strength, with absolute correlation coefficients exceeding 0.4.

In Dune genotypes, several significant correlations involved traits that share underlying measurements or derived relationships, such as SLWC–LT and associations linking LT or SLWC with SLA and LDMC (Figure 3); these associations are therefore not further interpreted as independent evidence of trait coordination. Among the remaining trait pairs, LDMC was positively correlated with SD (r = 0.488, *p* = 0.029) and I_1_ (r = 0.512, *p* = 0.021) at +1 °C. At +4 °C, SLA was negatively correlated with LDMC (r = −0.622, *p* = 0.003), whereas SD was positively correlated with I_1_ (r = 0.506, *p* = 0.023).

In Woods genotypes, all significant correlations under ambient conditions involved traits with shared underlying measurements or derived relationships and are therefore not further interpreted as independent evidence of trait coordination (Figure 3). At +1 °C, LT was positively correlated with SD (r = 0.527, *p* = 0.017), whereas SLA was negatively correlated with LDMC (r = −0.533, *p* = 0.015) and SD (r = −0.609, *p* = 0.004). At +4 °C, SLA was negatively correlated with LDMC (r = −0.500, *p* = 0.025) and SD (r = −0.521, *p* = 0.019).

Mantel tests, with BH-adjusted p-values, indicated significant similarity among correlation matrices, both between populations within the same temperature regime and within populations across temperature regimes. Dune and Woods correlation matrices were significantly similar under all three temperature regimes, while the Mantel correlation coefficients (r) were numerically lower at the higher temperature regimes, from ambient conditions (r = 0.752, *p* = 0.008) to +1 °C (r = 0.683, *p* = 0.004) and +4 °C (r = 0.600, *p* = 0.009). Within populations, Mantel correlation coefficients across temperature regimes were numerically higher than the between-population coefficients within the same temperature regime. In Woods genotypes, the correlation between +1 °C and +4 °C matrices was high (r = 0.967, *p* = 0.004). In Dune genotypes, correlation matrices were also significantly similar across temperature regimes, including the ambient and +4 °C comparison (r = 0.814, *p* = 0.011). Because differences among the Mantel coefficients themselves were not formally tested, numerical differences in coefficient magnitude are interpreted descriptively and do not demonstrate statistically significant differences in matrix similarity. In the reduced four-trait sensitivity analysis including SLA, LDMC, SD and I_1_, Mantel coefficients remained positive, but none of the between-population or within-population matrix comparisons remained significant after BH-adjustment (all *p* ≥ 0.125; Table S8B). Thus, the significant matrix similarities detected with the complete six-trait set were not robust to exclusion of SLWC and estimated LT.

Apart from the correlation analysis of individual functional and mechanistic leaf traits, each trait category was also analyzed as a composite trait group. Functional composite trait-group values were not significantly correlated across temperature regimes in Dune genotypes, whereas in Woods genotypes, they were significantly and positively correlated between +1 °C and +4 °C (r = 0.586, *p* = 0.007). The mechanistic composite trait group showed positive correlations across temperature regimes in both populations. In Dune genotypes, significant correlations were detected between ambient and +1 °C (r = 0.454, *p* = 0.045), ambient and +4 °C (r = 0.574, *p* = 0.008) and +1 °C and +4 °C temperature conditions (r = 0.611, *p* = 0.004). A similar pattern was observed in Woods genotypes, where mechanistic composite trait-group values were also significantly correlated between ambient and +1 °C (r = 0.624, *p* = 0.003), ambient and +4 °C (r = 0.691, *p* < 0.001) and +1 °C and +4 °C temperature conditions (r = 0.753, *p* < 0.001). In addition, the association between functional and mechanistic composite trait groups was examined within each population and temperature regime. In Dune genotypes, functional and mechanistic composite trait group values were negatively correlated under ambient temperature (r = −0.639, *p* = 0.002) and at +1 °C (r = −0.588, *p* = 0.006); by comparison, in Woods genotypes, significant negative correlations were consistently detected across all temperature regimes, under ambient (r = −0.478, *p* = 0.033), at +1 °C (r = −0.665, *p* = 0.001) and at +4 °C (r = −0.490, *p* = 0.029). Because the two composite trait groups include variables that share underlying measurements or derived relationships, these correlations are interpreted descriptively rather than as independent evidence of a functional trade-off between trait groups.

In the reduced four-trait sensitivity analysis, cross-temperature correlations within the mechanistic composite trait group, now based only on SD and I_1_, remained positive and significant in both populations (Dune: r = 0.467–0.532, *p* = 0.016–0.038; Woods: r = 0.498–0.581, *p* = 0.007–0.026; Table S8C). In contrast, no statistically significant correlations were detected between the functional and reduced mechanistic composite trait groups in any population × temperature-regime combination (Dune: r = 0.098–0.396, *p* = 0.084–0.680; Woods: r = −0.075–0.270, *p* = 0.250–0.754; Table S8D). Thus, the negative functional–mechanistic associations observed with the complete six-trait set were not robust to exclusion of SLWC and estimated LT. An additional sensitivity analysis using common min–max normalization across all three temperature regimes yielded numerically different correlation coefficients, but the pattern of statistical significance remained unchanged for all 30 comparisons (Table S9). In both the six-trait and four-trait analyses, cross-temperature correlations within the mechanistic composite remained positive and significant in both populations, whereas correlations between the functional and mechanistic composites remained nonsignificant in the reduced four-trait analysis.

Correlations among plasticity indices were examined across the three temperature contrasts (Table S5). Several significant associations involved plasticity indices of traits that share underlying measurements or derived relationships, particularly those involving SLWC and LT, and are therefore not interpreted as independent evidence of coordinated plasticity. Among the remaining trait pairs, *PI*_V_ SLA was positively correlated with *PI*_V_ LDMC in Woods genotypes under ambient vs. +4 °C contrast (ρ = 0.453, *p* = 0.045). Other correlations among plasticity indices were either non-significant or marginal and were not consistent across contrasts or populations.

To further evaluate the overall magnitude of leaf trait integration within the analyzed trait set, r*VE* values were examined for each temperature regime separately in Dune and Woods genotypes (Table S6). In both populations, r*VE* followed a similar pattern across temperature regimes, with the highest values at +1 °C and lower values under ambient and +4 °C conditions. However, omnibus permutation tests based on 10,000 permutations showed no significant differences in r*VE* among temperature regimes within either Dune (*p* = 0.458) or Woods (*p* = 0.141) genotypes. Between-population permutation tests likewise showed no significant differences under ambient (*p* = 0.217, BH-adjusted *p* = 0.650), +1 °C (*p* = 0.999, BH-adjusted *p* = 0.999), or +4 °C (*p* = 0.974, BH-adjusted *p* = 0.999) conditions. Because the omnibus permutation tests were not significant, no post hoc pairwise comparisons among temperature regimes were performed. Thus, no statistically detectable differences in the overall magnitude of phenotypic integration were found across temperature regimes or between populations. The reduced four-trait sensitivity analysis showed a somewhat different descriptive pattern of r*VE* values, but the inferential conclusion was unchanged. In Dune genotypes, r*VE* was highest at +1 °C, whereas in Woods genotypes, it increased from ambient to +4 °C (Table S8A). However, omnibus permutation tests detected no significant differences among temperature regimes within either Dune (*p* = 0.196) or Woods (*p* = 0.241) genotypes. Between-population differences were also nonsignificant under ambient (*p* = 0.855, BH-adjusted *p* = 0.959), +1 °C (*p* = 0.123, BH-adjusted *p* = 0.370), and +4 °C (*p* = 0.959, BH-adjusted *p* = 0.959) conditions. Thus, the conclusion that the overall magnitude of phenotypic integration did not differ detectably among temperature regimes or between populations was robust to exclusion of SLWC and estimated LT.

## 3. Discussion

### 3.1. Leaf Trait Responses to Warming

Phenotypic adjustment of leaf traits in response to changing thermal conditions can involve changes in leaf structure, resource-use and physiological functioning [7,33]. Our findings show that *I. pumila* adjusted both functional and mechanistic leaf traits across the temperature sequence. SLA, SLWC, estimated LT and I_1_ increased overall, whereas LDMC and SD declined, although the magnitude and progression of these changes differed among traits. Thus, changes across the warming sequence involved not only functional traits associated with leaf resource-use strategy [14], but also hydration–structural, stomatal–anatomical and chlorophyll-related components of the leaf phenotype.

Together, SLA and LDMC describe the functional direction of leaf adjustment. The gradual increase in SLA and the parallel decline in LDMC across the warming sequence point to a shift from a more conservative toward a more resource-acquisitive leaf strategy [13,14]. Higher SLA represents greater leaf area per unit dry mass, whereas lower LDMC indicates lower dry-mass investment relative to fresh mass. Within the leaf economics framework, the combination of higher SLA and lower LDMC is commonly associated with faster resource acquisition, higher potential growth rates and shorter tissue lifespan [13,14,34]. Taken together, these changes in SLA and LDMC are consistent with a shift in leaf resource-use strategy, but do not by themselves demonstrate increased carbon gain, improved whole-plant performance or adaptive benefit under warming. Increased SLA under experimental warming has also been reported in alpine plants, although other leaf-trait responses differed among species [35].

Changes in functional traits were already evident at +1 °C and became more pronounced across the full warming sequence. Both SLA and LDMC changed significantly between ambient conditions and +1 °C and showed further significant changes between +1 °C and +4 °C, indicating a progressive rather than sudden shift in the functional leaf phenotype. However, these functional traits do not by themselves identify the more specific structural and physiological components accompanying this shift. Functional and mechanistic traits can therefore provide complementary information for understanding plant responses to climate change [18,19]. In the present study, the mechanistic traits provide an additional level of interpretation by representing hydration–structural, stomatal–anatomical and chlorophyll-related components of the leaf phenotype. This functional–mechanistic distinction is used as a framework for interpreting complementary aspects of the leaf phenotype and does not imply independence among traits within or across the two categories.

The hydration–structural component of the response was represented by SLWC and LT. Both traits increased overall across the temperature sequence, although their trajectories differed between populations. By definition, SLWC expresses leaf water content per unit area and is considered a trait related to leaf water balance [18]. Its increase therefore indicates that the shift toward higher SLA and lower LDMC was not accompanied by a reduction in water content per unit leaf area. However, without direct measurements of leaf water potential or hydraulic status, higher SLWC should not be interpreted as evidence of improved plant water status or greater capacity to maintain leaf function under warming. Estimated LT showed the same overall direction of change as SLWC. However, the response trajectories of both SLWC and LT were population-dependent, and their detailed patterns are therefore considered below. Taken together, these results show that the functional shift in SLA and LDMC was accompanied by changes in the hydration–structural component of the leaf phenotype, without implying a specific anatomical or physiological mechanism.

The stomatal–anatomical component showed a different response pattern. SD did not change significantly during the initial increase from ambient temperature to +1 °C, whereas a significant decline occurred between +1 °C and +4 °C. This indicates that the stomatal-density response emerged later in the warming sequence than the changes in SLA and LDMC. Temperature effects on SD are not uniform across species, with decreases, increases and no response to elevated temperature reported under different experimental conditions [36]. A recent meta-analysis found an overall positive association between growth temperature and SD, but with only moderate consistency among experiments [37]. Because stomatal traits influence the anatomical potential for CO_2_ and water-vapor exchange, lower SD may alter the potential for gas exchange and water loss [38,39]. However, SD alone cannot be used to infer changes in stomatal conductance or transpiration, because gas exchange depends not only on stomatal density but also on stomatal size, aperture and other aspects of stomatal organization and behavior [36,38,39,40,41]. The observed decline should therefore be interpreted primarily as a change in stomatal anatomical organization across the temperature sequence, rather than as direct evidence of reduced water loss.

The chlorophyll-related component of the response was represented by the RGB-derived index I_1_, inversely related to chlorophyll content. I_1_ increased significantly already between ambient conditions and +1 °C and remained elevated at +4 °C, with no statistically detectable further change between the two elevated temperature regimes. A previous pilot experiment in *I. pumila* demonstrated a significant negative relationship between I_1_ values obtained from scanned leaves and chlorophyll concentration measured on the same leaves using the DMSO method [31,42]. Because chlorophyll concentration was not measured directly in the present study, changes in I_1_ are interpreted here as changes in a chlorophyll-related signal rather than as direct measurements of pigment concentration. Chloroplast processes, including chlorophyll biosynthesis and photochemical reactions, are sensitive to high temperature [43]. The early increase in I_1_ therefore indicates that the chlorophyll-related component of the leaf phenotype showed a detectable change already at +1 °C, whereas the absence of a further significant change suggests that this response did not increase progressively across the full warming sequence. A similar increase in I_1_ under +1 °C was previously observed in *I. pumila* [31], supporting the reproducibility of this early chlorophyll-related response to experimental warming. However, because I_1_ is an indirect optical indicator and photosynthetic responses involve multiple interacting processes that cannot be resolved without direct measurements such as gas exchange or chlorophyll fluorescence [44], its response should not be interpreted as direct evidence of altered photosynthetic performance.

Patterns of *PI*_V_ further showed that differences among traits were not consistently aligned with the functional versus mechanistic classification. Significant pairwise differences in *PI*_V_ were restricted to particular traits, populations and temperature contrasts. Such trait-specificity is consistent with reports that intraspecific trait variation can differ among leaf traits under experimental warming accompanied by reduced soil moisture [45]. In the present study, these patterns do not support a general conclusion that mechanistic traits were uniformly more plastic than functional traits. Instead, significant differences in *PI*_V_ involved particular trait pairs rather than a consistent functional-mechanistic pattern.

Overall, these results show a multi-trait pattern of phenotypic adjustment across the sequential temperature regimes, involving both functional and mechanistic leaf traits. Functional traits revealed a shift toward a more resource-acquisitive leaf strategy, whereas mechanistic traits reflected hydration–structural, stomatal–anatomical and chlorophyll-related components of the response. The different trajectories of these traits indicate that phenotypic adjustment in *I. pumila* across the warming sequence was not uniform but involved distinct response patterns among components of the leaf phenotype. Such heterogeneity is consistent with evidence that leaf morphological responses to warming can vary substantially among traits and taxa [46].

### 3.2. Population-Specific Patterns of Leaf Trait Responses to Warming

Although both populations showed changes in the same general direction, they differed in overall trait levels and in the dynamics of selected trait responses across the warming sequence.

The most persistent population differences were observed in the overall levels of SLA, LDMC and I_1_. Dune genotypes maintained higher SLA and I_1_ across temperature regimes, whereas Woods genotypes maintained higher LDMC. The combination of higher SLA and lower LDMC in Dune genotypes indicates a relatively more resource-acquisitive leaf phenotype, whereas the opposite pattern in Woods genotypes is consistent with a relatively more conservative resource-use strategy [13,14]. Higher I_1_ values in Dune genotypes, corresponding to a lower chlorophyll-related signal, further indicate a population difference in the chlorophyll-related component of the leaf phenotype. Because these differences were maintained under the same controlled growth conditions, they indicate population-associated differentiation in leaf-trait expression that cannot be attributed solely to the immediate experimental environment [47,48].

These population differences should be considered in the context of the contrasting ecological backgrounds of the two populations. Dune genotypes originate from an open, exposed habitat characterized by high irradiance and pronounced environmental fluctuations, whereas Woods genotypes originate from a shaded woodland habitat with a more buffered microclimate [26,29,30]. Because both populations had been maintained for several years under common growth-room conditions and were exposed to the same light environment during the present experiment, the observed differences cannot be attributed to immediate variation in irradiance. Instead, they may reflect persistent population-level differentiation in trait expression and environmental responsiveness [2,49]. Because only one population was sampled from each habitat type, the contribution of habitat origin cannot be separated from population identity. However, the specific direction of these trait differences should not be interpreted as a simple retained sun-shade phenotype, because leaf traits in *I. pumila* are themselves strongly plastic to light conditions [26].

The reaction norms further showed that differences between populations were not expressed in the same way across all traits. For SLA, LDMC, SD and I_1_, the response profiles of Dune and Woods genotypes were broadly parallel, indicating that the two populations showed largely similar response patterns for these traits. For SLA, LDMC and I_1_, differences between populations were therefore expressed mainly as persistent differences in overall trait levels, rather than through strongly divergent trajectories across the warming sequence. In contrast, SLWC and LT showed significant temperature × population interactions, indicating non-parallel response profiles between the two populations. These results show that the clearest population-specific differences involved the hydration–structural component of the leaf response.

SLWC increased between ambient and +1 °C in both populations, whereas a further increase between +1 °C and +4 °C was observed only in Woods genotypes. For LT, the increase was more pronounced between ambient and +1 °C in Dune genotypes, whereas Woods genotypes showed a gradual increase across the three regimes. These contrasting trajectories indicate that population differences were expressed mainly in the patterns of change in hydration–structural traits across successive temperature regimes rather than in opposite directions of response.

Stomatal density showed a different pattern from the hydration–structural traits. Although the two populations differed descriptively in the shape of their reaction norms, the absence of a significant temperature × population interaction indicates that these differences were not strong enough to support population-specific stomatal responses. Instead, SD appears to represent a broadly shared, later-emerging component of the response, as the main decline occurred only at the higher temperature increment. This pattern suggests that stomatal anatomical adjustment was more evident at the later stage of the temperature sequence (+4 °C) than at +1 °C. However, changes in SD should be interpreted cautiously because stomatal density alone does not determine gas exchange or transpiration; these processes also depend on stomatal size, aperture and other aspects of stomatal organization and behavior [36,38,39,40,41].

Patterns of *PI*_V_ showed that differences between the two populations were restricted to particular trait-contrast combinations. Dune genotypes exhibited higher *PI*_V_ for LDMC and SLWC early in the warming sequence, whereas Woods genotypes had higher LDMC *PI*_V_ for the later contrast (+1 °C vs. +4 °C). Thus, neither population showed consistently higher *PI*_V_ across traits and temperature contrasts. This context dependence is consistent with broader evidence that phenotypic plasticity among plant populations varies across traits and environmental conditions [27].

Patterns of within-population variability differed descriptively among traits, populations and temperature regimes. Functional traits generally showed lower variability than mechanistic traits, with I_1_ and SD among the most variable. A similarly high variability of I_1_ was observed in our previous warming experiment with *I. pumila* [31]. However, after accounting for the paired structure of the data and multiple testing, statistical support for changes in variance across temperature regimes was limited, with a significant difference detected only for SLA and a marginal difference for I_1_ in Dune genotypes. This suggests that the apparent shifts in trait dispersion across the temperature sequence were not accompanied by a broad restructuring of within-population variability.

Taken together, the two *I. pumila* populations differed mainly in overall trait levels and in the response patterns of selected leaf traits, accompanied by limited changes in within-population variance, rather than showing completely divergent responses across the warming sequence. SLA and LDMC reflected a broadly shared functional adjustment while maintaining population differences in trait means, whereas SLWC and LT showed population-dependent hydration–structural responses. Thus, the two populations differed in how particular components of the leaf response were expressed across the warming sequence, rather than showing entirely separate directions of response.

The present results extend our previous experimental warming study in *I. pumila*, in which a +1 °C increase in growth temperature induced trait-specific plastic responses in the same set of leaf traits [31]. The current study shows that responses expressed during the initial increase from ambient to +1 °C did not necessarily continue in the same manner as temperature increased further. Some traits changed across both temperature increments, others responded mainly during one increment, and SLWC and LT showed population-dependent response patterns. Thus, responses observed at +1 °C should not be assumed to predict the full pattern of leaf responses across a larger warming sequence.

These population-specific patterns do not demonstrate that either population is generally more plastic or better adapted to warming. Both Dune and Woods genotypes showed phenotypic adjustment across the temperature regimes, but population differences were concentrated in particular traits and temperature increments. The clearest population-specific differences involved the hydration–structural component, with a more pronounced initial LT increase in Dune genotypes and continued increases in SLWC and LT in Woods genotypes, suggesting distinct patterns of phenotypic adjustment across the temperature regimes. Such population-specific phenotypic responses should not be assumed to be adaptive without direct evidence of fitness consequences [6,10]. These findings therefore do not establish that either population performs better under warmer conditions.

### 3.3. Phenotypic Integration Under Warming

Leaf responses in *I. pumila* across the warming sequence were expressed not only through changes in individual trait values, but also through changes in the relationships among them. Such covariation is central to phenotypic integration, because traits forming a complex phenotype may be linked through shared functional, structural and developmental relationships [15,21]. This multivariate perspective is consistent with the view that plant responses reflect coordinated trait combinations rather than the behavior of isolated traits [20]. Phenotypic integration has previously been examined in *I. pumila* using floral traits, providing a species-specific basis for analyzing patterns of trait covariation [50]. Environmental change can modify these relationships, so variation in trait correlations may provide information on how the organization of the leaf phenotype changes under altered thermal conditions [21,23]. Recent experimental evidence further shows that environmental conditions can modify intraspecific covariation among leaf economic traits [24].

Trait correlations revealed significant associations in both populations under all temperature regimes, but their number, direction and distribution varied between Dune and Woods genotypes and across temperatures. Descriptively, these findings suggest variation in both individual trait expression and patterns of relationships among leaf traits across populations and temperature regimes.

The most consistent association was the positive association between SLWC and LT, which was detected in both populations under all three temperature regimes. However, because these traits are not mathematically independent, the strength and persistence of this association may partly reflect shared underlying measurements and should therefore not be interpreted as fully independent evidence of biological trait coordination. Associations involving SLA, LDMC, SD and I_1_ varied more across populations and temperature regimes, indicating greater context dependence in these relationships. Such context dependence is consistent with evidence that relationships among leaf economic traits may vary across local environmental conditions rather than remaining fixed [51].

In Woods genotypes, elevated temperature was accompanied by persistent negative associations of SLA with LDMC and SD. In Dune genotypes, the pattern of interpretable correlations was more variable across temperature regimes. Descriptively, selected trait associations were detected more consistently across the elevated temperature regimes in Woods genotypes, whereas the pattern of significant associations was more variable across temperature regimes in Dune genotypes. Together with the consistently lower SLA and higher LDMC of Woods genotypes, this pattern is compatible with a relatively more conservative leaf strategy across the warming sequence. Such differences are in line with evidence that responses to climatic warming can vary among populations and depend on ecological context [52,53].

The six-trait Mantel analyses indicated significant similarities in trait-correlation structure both between populations at each temperature regime and within each population across temperature regimes. However, these similarities were not retained in the reduced four-trait sensitivity analysis: although Mantel coefficients remained positive, none of the comparisons remained significant after BH adjustment. Thus, the evidence for similarity of the overall correlation-matrix structure was not robust to exclusion of SLWC and estimated LT and should therefore be interpreted cautiously. Likewise, the numerically lower between-population Mantel coefficients at the higher temperature regimes in the six-trait analysis are treated as descriptive differences rather than evidence of a statistically demonstrated decline in matrix similarity. Taken together, these results indicate descriptive variation in trait-correlation structure across the warming sequence, while the evidence for retention of the overall matrix structure was sensitive to the composition of the trait set.

At the trait-group level, cross-temperature associations within the mechanistic composite trait group were positive across temperature comparisons in both populations. This pattern persisted in the reduced four-trait sensitivity analysis, in which the mechanistic composite trait group comprised only SD and I_1_, indicating that the maintenance of positive cross-temperature covariation within this trait group did not depend on the inclusion of SLWC and estimated LT. In contrast, relationships within the functional composite trait group were more variable across temperature comparisons, particularly in Dune genotypes. Thus, the two composite trait groups differed in the stability of their covariation across temperature regimes, with the mechanistic group showing a more persistent cross-temperature pattern.

Relationships between the functional and mechanistic composite trait groups were generally negative in the complete six-trait analysis, although their statistical expression differed between populations and temperature regimes. However, these associations were not retained in the reduced four-trait sensitivity analysis: correlations between the functional composite and the reduced mechanistic composite trait groups were nonsignificant in all population × temperature-regime combinations. Because estimated LT is derived from SLA and LDMC and SLWC shares the same underlying gravimetric measurements, part of the negative covariation observed in the complete trait set was structurally expected. The loss of these associations after exclusion of SLWC and estimated LT therefore indicates that the negative functional–mechanistic pattern was sensitive to the mathematical dependence among traits and should not be interpreted as independent evidence of a functional trade-off. The persistence of the same pattern of statistical significance under common min–max normalization across temperature regimes further suggests that the qualitative conclusions concerning composite trait-group associations were not dependent on the choice of normalization, despite numerical changes in correlation coefficients (Table S9).

Correlations among *PI*_V_ values showed that phenotypic changes in different leaf traits were themselves associated, although most relationships depended on population and temperature contrast. Such associations may reflect shared environmental sensitivity, functional relationships or common genetic control among traits [23,26,54]. The most consistent association in the present study was between *PI*_V_ values for SLWC and LT, which was detected in both populations across all temperature contrasts. A similarly strong positive association between SLWC and LT plasticity was previously observed in *I. pumila* under experimental warming [31]. Given the non-independence of SLWC and LT, however, the consistency of this association should be interpreted cautiously and not as fully independent evidence of coordinated plasticity. Other correlations among *PI*_V_ values were population- or contrast-specific, indicating that associations among phenotypic changes were concentrated in particular trait combinations rather than expressed uniformly across the leaf phenotype. This pattern is consistent with evidence that phenotypic integration and plasticity are linked through covariation among plastic responses rather than through a simple relationship between the overall magnitude of plasticity and integration [25].

The r*VE* analysis provided a complementary perspective on the overall magnitude of phenotypic integration. In the complete six-trait analysis, r*VE* values were highest at +1 °C in both populations, but permutation tests detected no significant differences among temperature regimes or between populations. Given the mathematical dependence of SLWC and estimated LT on SLA and LDMC, greater interpretive weight was placed on the reduced four-trait analysis when evaluating the overall magnitude of phenotypic integration. The reduced four-trait sensitivity analysis produced a different descriptive pattern, particularly in Woods genotypes, but likewise detected no significant differences among temperature regimes or between populations. Thus, the absence of statistically detectable differences in the overall magnitude of phenotypic integration was consistent across both trait sets, although these nonsignificant findings do not establish that phenotypic integration remained unchanged. This result contrasts with the clearer population- and temperature-dependent variation observed in particular trait relationships, indicating that changes in the pattern of covariation across the temperature sequence did not necessarily translate into detectable changes in its overall magnitude [22,55]. Because the complete trait set includes mathematically related variables, r*VE* from that analysis is interpreted as a summary of covariation within the analyzed trait set rather than as a measure of integration among fully independent traits.

The sequential design should be considered when interpreting the patterns described above. Because the temperature regimes were imposed sequentially in a single growth room, chronological time necessarily covaried with the temperature sequence and possible carry-over effects from the preceding regime cannot be excluded. Although leaf developmental stage was standardized by sampling newly developed, fully expanded leaves, the genotypes represent repeated biological observations rather than independent replication of the temperature regimes.

Taken together, these results indicate that leaf responses across the warming sequence involved changes not only in individual leaf traits but also in particular patterns of trait covariation. Some of these patterns differed between populations, although the sensitivity analyses showed that conclusions about the overall correlation-matrix structure depended on the composition of the trait set. Predicting the response of *I. pumila* to future warming may therefore benefit from considering trait expression, phenotypic plasticity and trait covariation together, while accounting for the dependence of multivariate patterns on the traits included in the analysis.

## 4. Materials and Methods

### 4.1. The Study Species

*Iris pumila* L. (Iridaceae) is a rhizomatous perennial clonal monocot native to the Eurasian steppe region. Its range extends from Austria across central and southeastern Europe and western Siberia [56,57,58,59]. In Serbia, the species is native to the Deliblato Sands (44°57′59″ N, 21°01′53″ E), a large dune complex in the southeastern Pannonian Plain, where it inhabits both open dunes and shaded woodlands [60]. Dune populations occur in open sandy sites dominated by annual and perennial herbs and low shrubs, whereas woodland populations are associated with afforested areas dominated primarily by black pine (*Pinus nigra*), Scots pine (*P. sylvestris*) and black locust (*Robinia pseudoacacia*). These habitats differ markedly in abiotic conditions, especially in light intensity and light quality [29,30]. Open dune habitats are exposed to high irradiance and pronounced temperature fluctuations, whereas woodland habitats are characterized by a more buffered microclimate [29,30]. In *I. pumila*, individual vegetative modules (ramets) of the same genotype are interconnected by horizontally growing rhizomes and form compact, circular clones. Because vegetative propagation through rhizomes allows the same genotype to persist for many years, this species is well suited for experimental studies of phenotypic plasticity and genotype-specific responses to environmental variation [61].

### 4.2. Experimental Setup, Leaf Sampling and Leaf Trait Measurement

The plants used in this study originated from two natural populations of *I. pumila* inhabiting contrasting light habitats in the Deliblato Sands: an open sandy dune population, hereafter referred to as Dune, and a shaded woodland population, hereafter referred to as Woods. Twenty distinct clonal genotypes were selected from each population, giving a total of 40 genotypes. Prior to the experimental warming regimes, rhizome modules from both populations were maintained under controlled growth-room conditions for three years to minimize maternal environmental effects and to standardize the physiological state of the experimental material. Plants were grown individually in 500 cm^3^ pots containing a 2:1 (*v*/*v*) mixture of soil substrate and sand. They were maintained in a growth room at 23/19 °C day/night under a 16 h photoperiod, with photosynthetic photon flux density (PPFD) of 110.5 µmol m^−2^ s^−1^. The applied PPFD reflected the maximum output of the available growth-room lighting system and was held constant across all temperature regimes. Plants were watered once weekly, with a diluted commercial microbiological fertilizer (Slavol, Agrounik d.o.o., Šimanovci, Serbia), described by the manufacturer as containing nitrogen-fixing and phosphomineralizing bacteria, and prepared at 10 mL L^−1^ of water. Soil moisture was monitored using a ThetaProbe ML2x soil moisture sensor (Delta-T Devices Ltd., Cambridge, UK) and watering volume was adjusted under the +4 °C regime to minimize substrate drying at the higher temperature. Pots were randomly arranged on growth-room shelves and rotated twice weekly to reduce positional effects.

Relative to these ambient conditions, the +1 °C and +4 °C regimes were selected to represent moderate and stronger increases in growth temperature within the range of projected global temperature increases during this century [1]. The three temperature regimes were imposed sequentially in the same growth room, while photoperiod, light source, substrate, watering schedule and pot rotation were maintained consistently throughout the experiment. Plants were initially maintained under ambient conditions and fully expanded leaves that had developed under this temperature regime were sampled. The temperature was then increased by 1 °C (24/20 °C day/night). After approximately one month, when newly developed leaves had fully expanded under the +1 °C regime, these leaves were sampled from the same genotypes. Following this sampling, temperature was further increased to +4 °C relative to ambient (27/23 °C day/night) and maintained for approximately one month, after which newly developed, fully expanded leaves were sampled. Thus, the same clonal genotypes were followed through the sequence of temperature regimes and leaf traits were measured on leaves developed under each respective temperature regime. Accordingly, responses at +4 °C were measured following prior exposure to the +1 °C regime rather than responses of plants transferred directly from ambient to +4 °C.

Because leaf developmental stage and position along the leaf blade can influence trait values, all measurements were performed on the upper two-thirds of the last fully expanded leaf. At each temperature regime, one such leaf was sampled from the same ramet of each genotype and fresh mass was measured immediately after excision. Leaf area was then recorded from digital images obtained using an optical scanner (Epson Perfection V600, Epson, Suwa, Japan) at a resolution of 600 dpi in 24-bit RGB color mode and projected leaf area was determined using ImageJ software (v1.51j8) [62]. After scanning, leaf samples were oven-dried at 60 °C for 72 h to constant mass and their dry mass was weighed.

Phenotypic values of the analyzed leaf traits were obtained following the protocols used in our previous study [31], with trait calculations based on standardized plant trait measurement procedures [63]. Specific leaf area (SLA, cm^2^ g^−1^) was calculated as leaf area divided by leaf dry mass, leaf dry matter content (LDMC, g g^−1^) as leaf dry mass divided by fresh mass, and specific leaf water content (SLWC, g cm^−2^) as the difference between fresh and dry mass divided by leaf area. Leaf thickness (LT, cm) was estimated following Vile et al. [32] from (SLA × LDMC)^−1^, under the approximation of a leaf fresh-mass density close to 1 g cm^−3^; more explicitly, LT = (1/ρF)(SLA × LDMC)^−1^, where ρF is the apparent leaf density on a fresh-mass basis. Stomatal density (SD) was estimated using the epidermal impression method, as described previously [64]. Briefly, transparent nail polish impressions were taken from the middle section of the leaf surface, placed on microscope slides and stomata were counted in 10 randomly selected microscopic fields per sample using a Leica light microscope (Leica, Wetzlar, Germany) with a 20× objective. Each field covered 466 × 350 µm (0.163 mm^2^). For each sample, the stomatal counts from the 10 fields were averaged, and stomatal density was calculated by dividing this mean count by the field area and expressed as number of stomata mm^−2^. Because *I. pumila* has amphistomatous, vertically oriented leaves with no clear distinction between adaxial and abaxial surfaces [65], the same arbitrarily selected leaf side was used for all samples to ensure consistency.

RGB values were extracted from the scanned leaf images from five circular regions of identical area positioned at different locations on each leaf. The same scanner settings, image-processing procedures and RGB extraction methods were used for all samples across the three temperature regimes. Mean R, G and B values were obtained from all pixels within each region and averaged across the five regions for each leaf. The RGB index I_1_ was then calculated as I_1_ = R + G − 2B, where R, G and B represent the red, green and blue image components, respectively, following Sánchez-Sastre et al. [66]. Its use as a chlorophyll-related indicator in *I. pumila* is supported by a previous species-specific calibration [31], in which I_1_ values obtained from scanned leaves showed a significant negative relationship with total chlorophyll concentration measured on the same leaves using the DMSO method [42] (n = 30, r = −0.631, *p* = 0.0002). Accordingly, higher I_1_ values were interpreted as indicating a lower chlorophyll-related signal.

### 4.3. Statistical Analyses 

All statistical analyses were performed at the genotype level, with each genotype providing repeated biological observations across the temperature sequence. Because the temperature regimes were imposed sequentially in a single growth room, the genotypes did not constitute independent replicates of the temperature regimes themselves. A total of 40 clonal genotypes were analyzed, with 20 originating from the Dune population and 20 from the Woods population. Descriptive statistics, including means, standard errors and coefficients of variation, were calculated for each leaf trait, population and temperature regime using the PROC MEANS procedure in SAS 9.3 [67]. Differences in phenotypic variance across temperature regimes within each population were evaluated using paired Pitman–Morgan tests, thereby accounting for repeated measurements of the same genotypes [68]. Bivariate normality was assessed separately for each paired comparison using the Henze–Zirkler test [69]. For each trait within each population, the three pairwise temperature comparisons (ambient vs. +1 °C, ambient vs. +4 °C, and +1 °C vs. +4 °C) were treated as one family of hypotheses, and *p*-values were adjusted using the Holm procedure. Between-population differences in variance were evaluated separately within each temperature regime using two-sided *F*-tests for equality of variances. Normality was assessed separately for each population × temperature-regime combination using the Shapiro–Wilk test. For each trait, the three Dune–Woods comparisons across temperature regimes were treated as one family of hypotheses, and p-values were likewise adjusted using the Holm procedure. All variance tests were two-sided, with the alternative hypothesis of unequal variances. The Pitman–Morgan tests had df = 18, whereas the *F*-tests had df_1_ = 19 and df_2_ = 19. The Pitman–Morgan tests were performed using the *PairedData* package (version 1.1.1) [70], and the Henze–Zirkler tests using the *mvnTest* package (version 1.1-0) [71]. The *F*-tests, Shapiro–Wilk tests, and Holm adjustments were performed using functions from the base R *stats* package (version 4.6.1). Reaction norm plots were generated for each genotype across the three temperature regimes using GraphPad Prism [version 8; GraphPad Software, San Diego, CA, USA].

To assess variation in individual leaf traits, each trait was analyzed separately using repeated-measures analysis of variance, including temperature regime, population and their interaction. Temperature regime, with three levels (ambient, +1 °C and +4 °C), was treated as the within-subject factor, because the same clonal genotypes were followed across all three regimes. Population, with two levels (Dune and Woods), was included as the between-subject factor. Normality of residuals and homogeneity of variances were checked before analysis. Within-subject effects were evaluated using multivariate tests based on Wilks’ λ. The multivariate approach was used because it accounts for the covariance structure among repeated measurements without requiring the sphericity assumption; consequently, the Greenhouse–Geisser correction was not applied [72]. For significant population effects, effect size was expressed as partial eta squared (ηp^2^), calculated from the corresponding *F* statistic and degrees of freedom.

The repeated-measures design was further interpreted within the framework of profile analysis [72]. The temperature × population interaction tested whether the shape of the response profiles differed between Dune and Woods genotypes. A significant interaction was interpreted as evidence of differences in response profiles between populations across the temperature sequence. Simple-effect comparisons were subsequently used to examine changes across temperature regimes within each population and population differences within individual temperature regimes. In the absence of a significant temperature × population interaction, the main effect of population was interpreted as a difference in overall profile level, whereas the main effect of temperature indicated deviation from profile flatness. Profile contrasts were used to compare successive temperature increments (ambient vs. +1 °C and +1 °C vs. +4 °C) and the overall ambient vs. +4 °C contrast. For each contrast, the population × contrast effect was examined to determine whether the magnitude of change across the corresponding temperature contrast differed between populations. Profile analysis was performed using PROC GLM with the REPEATED/PROFILE option in SAS [67]. Although repeated-measures ANOVA accounts for the non-independence of observations from the same genotypes, it does not separate the effects of temperature from chronological time and possible carry-over effects in the sequential experimental design.

Phenotypic plasticity was analyzed separately from individual leaf trait values and was quantified for each genotype using a relative-distance measure, hereafter referred to as the phenotypic plasticity index (*PI*_V_), following the relative-distance approach of Valladares et al. [73]: *PI*_V_ = |X_1_ − X_2_|/(X_1_ + X_2_), where X_1_ and X_2_ represent trait values of the same genotype expressed under two different temperature regimes. Higher *PI*_V_ values indicate greater relative phenotypic difference between the two temperature regimes. *PI*_V_ was calculated separately for ambient vs. +1 °C, ambient vs. +4 °C and +1 °C vs. +4 °C contrasts. Because these contrasts differ in both temperature increment and position within the sequential experiment, differences in *PI*_V_ among contrasts were interpreted as differences in the magnitude of observed phenotypic change, rather than as standardized measures of responsiveness per unit temperature change. Higher *PI*_V_ values for a particular contrast were therefore not interpreted as evidence of inherently greater plasticity within that temperature interval or of an adaptive response.

Because plasticity indices did not meet the assumptions required for parametric analyses, nonparametric tests were used. Differences in plasticity among traits were tested separately for each population and temperature contrast using Friedman ANOVA in R (version 4.6.1) [74]. Each population × temperature-contrast combination was treated as a separate analysis. When the Friedman test was significant, the 15 possible pairwise comparisons among the six traits were performed using Wilcoxon signed-rank tests. The 15 post hoc comparisons within each population × temperature-contrast combination were treated as one family of hypotheses, and the Holm adjustment was applied within that family to control the family-wise error rate. For each trait and temperature contrast, differences in plasticity between Dune and Woods genotypes were tested using Mann–Whitney U tests, with *p*-values adjusted within each trait using the Benjamini–Hochberg false discovery rate procedure. Differences in *PI*_V_ among temperature contrasts were then tested separately for each trait within each population using Friedman ANOVA because the three *PI*_V_ values were calculated from shared trait measurements of the same genotypes and were therefore mathematically dependent. Significant effects were followed by pairwise Wilcoxon signed-rank tests with Holm adjustment applied separately within each trait × population combination. To evaluate the effects of population, temperature contrast and their interaction on *PI*_V_, a nonparametric longitudinal analysis was performed using the *nparLD* package (version 2.3.1) in R [75]. Population was treated as the between-subject factor and temperature contrast as the repeated factor, and effects were evaluated using an ANOVA-type statistic (ATS). Correlations among genotype-specific *PI*_V_ values were calculated separately for each population and temperature contrast using Spearman’s rank correlation coefficient (ρ). Phenotypic correlations among individual leaf traits were calculated separately for each population and temperature regime using Pearson’s correlation coefficients. Pearson and Spearman correlation analyses were treated as exploratory. No multiple-testing adjustment was applied, and the resulting unadjusted p-values were interpreted descriptively.

In addition to analyses of individual leaf traits, the functional and mechanistic trait categories were also analyzed as composite trait groups. To construct the composite trait-group values, each trait was min–max normalized separately within each temperature regime across all genotypes from both populations, thereby placing traits on a common 0–1 scale within each temperature regime. Normalization was performed according to the formula: Xnorm = (X − Xmin)/(Xmax − Xmin), where Xmin and Xmax are the minimum and maximum values of the respective trait within that temperature regime. The aim of this regime-specific normalization was to characterize relative variation among genotypes within each experimental regime, while ensuring that traits measured on different scales contributed comparably to the composite values. The functional trait group included normalized values of SLA and LDMC, whereas the mechanistic trait group included normalized values of SLWC, LT, SD and I_1_. For each genotype and temperature regime, the mean normalized value of all traits within each category was calculated to obtain a composite trait-group value. Correlations of trait-group values across temperature regimes were examined separately within each population, whereas correlations between functional and mechanistic trait-group values were examined within each population and temperature regime. To assess whether the use of different reference ranges across temperature regimes influenced these correlations, we performed an additional sensitivity analysis using a common minimum and maximum for each trait, calculated across all genotypes from both populations and all three temperature regimes. Composite trait-group values and their Pearson correlations were recalculated using this common normalization for both the complete six-trait set and the reduced four-trait set (SLA, LDMC, SD and I_1_). Similarity among the individual-trait correlation matrices was evaluated using Mantel tests implemented in the *vegan* package (version 2.6-4) in R [76]. Correlation matrices were converted to dissimilarity matrices as 1 − r, and Pearson Mantel correlations were calculated. Trait labels in one matrix were permuted by jointly reordering the corresponding rows and columns while the other matrix remained fixed [77]. Comparisons were performed among temperature regimes within each population and between populations within each temperature regime. Because the trait matrices contained six traits, there were 6! = 720 possible trait-label arrangements. Significance was therefore assessed by exhaustive enumeration of the complete permutation space: all 719 non-identity permutations were evaluated, with the observed arrangement completing the full set of 720 configurations [77,78]. The resulting *p*-values were adjusted using the Benjamini–Hochberg procedure. For each temperature regime, correlation matrices of the Dune and Woods populations were visualized as separate heat maps using the *corrplot* package (version 0.92) in R [79].

Phenotypic integration among the analyzed leaf traits was quantified using the relative variance of eigenvalues (r*VE*), following Pavličev et al. [55]. For each population and temperature regime, principal component analysis (PCA) was performed on the six standardized leaf traits using the *prcomp*() function in R. r*VE* was calculated from the full set of PCA eigenvalues. Eigenvalue variance was calculated as *VE* = Σ(λ_i_ − 1)^2^/N, where N is the number of traits, and relative eigenvalue variance was calculated as r*VE* = *VE*/(N − 1). Thus, r*VE* ranges from 0 to 1, with higher values indicating a greater overall magnitude of trait integration. Bootstrap resampling of genotypes with replacement within each population × temperature regime combination (10,000 iterations) was used to estimate 95% confidence intervals. Differences in r*VE* among temperature regimes within each population were assessed using an omnibus permutation test based on the variance among temperature regime-specific r*VE* values (10,000 permutations). Temperature-regime labels were permuted within genotype, while the complete trait vector was retained as a unit, thereby preserving both the repeated-measures structure and the covariance among traits. Differences between populations were evaluated separately within each temperature regime using permutation tests (10,000 permutations), in which population labels were randomly reassigned among genotypes while retaining the complete trait vector of each genotype. Resulting *p*-values were adjusted using the Benjamini–Hochberg procedure. Analyses were performed in R [74] and data processing was conducted using the *dplyr* package (version 1.1.4) [80].

To assess the influence of mathematical dependence among traits on the multivariate results, a sensitivity analysis was conducted excluding SLWC and estimated LT. The PCA, r*VE*, Mantel and composite trait-group analyses were repeated using only SLA, LDMC, SD and I_1_. For the four-trait Mantel analysis, the same exhaustive permutation procedure was used; the complete permutation space consisted of 4! = 24 configurations, comprising 23 non-identity permutations plus the observed arrangement. In the reduced composite analysis, the functional trait group remained defined by SLA and LDMC, whereas the mechanistic trait group included only SD and I_1_. Results from the reduced four-trait analyses were compared with those obtained from the complete six-trait set to assess the robustness of the main conclusions.

## 5. Conclusions

The present study showed trait-specific changes in functional and mechanistic leaf traits across the sequential temperature regimes in *I. pumila*, with population-specific responses evident for selected traits. Across the warming sequence, SLA increased and LDMC decreased, consistent with a shift toward a more resource-acquisitive leaf strategy. This functional adjustment was accompanied by changes in hydration–structural, stomatal–anatomical and chlorophyll-related components of the leaf phenotype.

The inclusion of two successive temperature increments showed that responses were not uniform across the warming sequence. Some changes were already expressed during the initial increase from ambient temperature to +1 °C, whereas others emerged or continued during the subsequent increase to +4 °C. SLA and LDMC changed progressively across both temperature increments, I_1_ responded mainly during the initial increase, and the main decline in SD occurred later. SLWC and LT showed population-specific patterns across the temperature regimes, indicating that trait patterns observed at +1 °C did not necessarily correspond to those observed at +4 °C.

The two populations originating from contrasting light habitats differed in both trait expression and the progression of selected responses. Dune genotypes maintained higher SLA and I_1_, whereas Woods genotypes maintained higher LDMC across temperature regimes. However, the two populations responded across the warming sequence in broadly similar directions, and population differences in plasticity were restricted to particular traits and temperature contrasts. The clearest population-specific responses involved SLWC and LT, with SLWC increasing initially in both populations but continuing to increase only in Woods genotypes, whereas LT increased more prominently between ambient and +1 °C in Dune genotypes and between +1 °C and +4 °C in Woods genotypes. The two populations therefore differed in when and how particular components of the leaf response were expressed without indicating that either population was generally more plastic or showed a superior response to warming.

Trait-correlation analyses provided a complementary view of these leaf responses. Across the warming sequence, particular patterns of trait covariation varied between populations and temperature regimes, while the sensitivity analysis showed that evidence for similarity of the overall correlation-matrix structure depended on the composition of the trait set. In contrast, no statistically detectable differences in the overall magnitude of phenotypic integration, quantified by r*VE*, were found among temperature regimes or between populations, and this conclusion was retained after exclusion of SLWC and estimated LT. Thus, variation in particular trait relationships across the warming sequence was not accompanied by a detectable change in the overall magnitude of phenotypic integration.

Considered together, trait means, phenotypic plasticity and patterns of covariation showed that leaf responses involved coordinated but nonuniform patterns across traits, temperature increments and population origins. Because the temperature regimes were imposed sequentially, chronological time covaried with the temperature sequence and possible carry-over effects from the preceding regime cannot be excluded. Accordingly, responses observed at +4 °C should be interpreted in the context of prior exposure to +1 °C, rather than as reflecting an independent transition from ambient to +4 °C.

## Figures and Tables

**Table 1:** Mean values (±SE) for the functional and mechanistic leaf traits: specific leaf area (SLA, cm^2^ g^−^^1^), leaf dry matter content (LDMC, g g^−^^1^), specific leaf water content (SLWC, g cm^−^^2^), leaf thickness (LT, cm), stomatal density (SD, stomata mm^−2^) and RGB-derived index I_1_, in *I. pumila* genotypes originating from two natural populations, Dune and Woods. A total of 40 genotypes (20 per population) were analyzed across sequential temperature regimes (ambient, +1 °C and +4 °C) in an environmentally controlled growth room. LT represents estimated leaf thickness derived from SLA and LDMC following Vile et al. [32].

Trait	Dune			Woods		
	Ambient	+1 °C	+4 °C	Ambient	+1 °C	+4 °C
*Functional*						
SLA	187.0 ± 2.0	189.0 ± 2.7	203.6 ± 4.2	167.9 ± 3.1	175.0 ± 3.5	182.4 ± 4.3
LDMC	0.2153 ± 0.0022	0.1909 ± 0.0030	0.1830 ± 0.0036	0.2264 ± 0.0032	0.2089 ± 0.0030	0.1902 ± 0.0037
*Mechanistic*						
SLWC	0.0197 ± 0.0003	0.0226 ± 0.0005	0.0225 ± 0.0004	0.0201 ± 0.0005	0.0213 ± 0.0004	0.0231 ± 0.0006
LT	0.0251 ± 0.0003	0.0279 ± 0.0005	0.0275 ± 0.0004	0.0260 ± 0.0006	0.0268 ± 0.0005	0.0285 ± 0.0007
SD	89.3 ± 3.5	92.8 ± 3.2	78.8 ± 2.5	93.1 ± 3.2	92.5 ± 3.3	85.1 ± 2.5
I_1_	143.0 ± 3.5	155.8 ± 5.3	163.5 ± 4.9	121.6 ± 5.0	132.2 ± 5.6	137.2 ± 5.4

**Table 2:** Repeated-measures ANOVA for functional and mechanistic leaf traits of *I. pumila* genotypes from Dune and Woods populations analyzed across sequential temperature regimes (ambient, +1 °C and +4 °C). Within-subject effects were evaluated using Wilks’ λ. Statistically significant *F*-values (*p* < 0.05) are shown in bold. For trait acronyms and measurement units see Table 1.

Between-Subjects	SLA			LDMC			SLWC			LT			SD			I_1_		
Source of variation	df	*F*	*p*	df	*F*	*p*	df	*F*	*p*	df	*F*	*p*	df	*F*	*p*	df	*F*	*p*
Population (P)	1	**23.77**	<0.0001	1	**13.01**	0.0009	1	0.03	0.8573	1	1.21	0.2783	1	0.93	0.3415	1	**17.33**	0.0002
Error	38			38			38			38			38			38		
**Within-Subjects**	Temperature									Temperature x Population								
Trait	Wilks’*λ*		*F*	Num df		Den df		*p*		Wilks’*λ*		*F*		Num df		Den df		*p*
**SLA**	0.576		**13.62**	2		37		<0.0001		0.943		1.12		2		37		0.3382
**LDMC**	0.195		**76.45**	2		37		<0.0001		0.862		2.97		2		37		0.0635
**SLWC**	0.283		**46.87**	2		37		<0.0001		0.774		**5.41**		2		37		0.0087
**LT**	0.475		**20.41**	2		37		<0.0001		0.786		**5.03**		2		37		0.0117
**SD**	0.485		**19.67**	2		37		<0.0001		0.932		1.35		2		37		0.2726
**I_1_**	0.552		**14.99**	2		37		<0.0001		0.986		0.26		2		37		0.7760

## Data Availability

The original contributions presented in this study are included in the article/Supplementary Materials. Further inquiries can be directed to the corresponding author.

## Supplementary Materials

The following supporting information can be downloaded at: https://www.mdpi.com/article/10.3390/plants15213292/s1, Table S1: Analyses of variance for each contrast of the within-subject factor, temperature regime, for functional and mechanistic leaf traits of *I. pumila* genotypes from Dune and Woods populations analyzed across sequential temperature regimes (ambient, +1 °C and +4 °C); Table S2: Simple-effect comparisons for SLWC and estimated LT in *I. pumila* genotypes from Dune and Woods populations analyzed across sequential temperature regimes (ambient, +1 °C and +4 °C); Table S3: Coefficient of variation (CV%) for the functional and mechanistic leaf traits in *I. pumila* genotypes originating from the Dune and Woods populations; Table S4: Comparisons of phenotypic variances in *I. pumila* genotypes within populations across temperature regimes and between populations within each temperature regime; Table S5: Spearman’s correlation coefficients (ρ) and *p*-values for plasticity indices (*PI*_V_) of *I. pumila* genotypes originating from Dune and Woods populations across three temperature contrasts; Table S6: Eigenvector coefficients of functional and mechanistic leaf traits for the first three principal components (PC1-PC3) in *I. pumila* genotypes originating from the Dune and Woods populations across sequential temperature regimes; Table S7: Results of nonparametric longitudinal analysis of plasticity indices (*PI*_V_) for functional and mechanistic leaf traits of *I. pumila* genotypes originating from Dune and Woods populations across three temperature contrasts; Table S8: Sensitivity analyses of trait covariance and phenotypic integration after exclusion of SLWC and estimated LT, using the reduced four-trait set comprising SLA, LDMC, SD and I_1_; Table S9: Sensitivity analysis of Pearson correlations involving functional and mechanistic composite trait groups using regime-specific and common min–max normalization.
