# Supplementary material for: Intraspecific Variation in Leaf Trait Responses to Experimental Warming in Iris pumila Populations from Contrasting Habitats
Source: Plants (Basel). 2026 Oct 25;15(21):3292. doi: 10.3390/plants15213292 (PMC13765610; doi:10.3390/plants15213292)
Supplement: Supplementary file 1 [file plants-15-03292-s001.zip › plants-4595427-supplementary.pdf]

**Table S1.** Analyses of variance for each contrast of the within-subject factor, temperature regime, for functional and mechanistic leaf traits of *I. pumila* genotypes from Dune and Woods populations analyzed across sequential temperature regimes (ambient, +1 °C and +4 °C). The contrast variables refer to ambient vs. +1 °C, +1 °C vs. +4 °C and ambient vs. +4 °C comparisons. Mean tests the overall contrast across populations, while Population tests whether the contrast differs between Dune and Woods genotypes. The three profile contrasts were treated as planned comparisons, and no additional multiple-testing adjustment was applied; the reported *p*-values are unadjusted. Significant *F*-values are shown in bold, while marginally significant results (0.05 < *p* < 0.10) are underlined. For trait acronyms and measurement units, see Table 1.

| Trait | | **SLA** | | | **LDMC** | | | **SD** | | | **$I_1$** | |
|---|---|---|---|---|---|---|---|---|---|---|---|---|
| Source of variation | df | *F* | *p* | df | *F* | *p* | df | *F* | *p* | df | *F* | *p* |
| *Contrast variable: ambient – +1 °C* | | | | | | | | | | | | |
| Mean | 1 | **4.71** | 0.0363 | 1 | **105.48** | <.0001 | 1 | 0.29 | 0.5948 | 1 | **12.23** | 0.0012 |
| Population | 1 | 1.46 | 0.2347 | 1 | <u>2.87</u> | 0.0985 | 1 | 0.57 | 0.4559 | 1 | 0.11 | 0.7390 |
| Error | 38 | | | 38 | | | 38 | | | 38 | | |
| *Contrast variable: +1 °C – +4 °C* | | | | | | | | | | | | |
| Mean | 1 | **14.78** | 0.0004 | 1 | **25.94** | <.0001 | 1 | **28.29** | <.0001 | 1 | 2.39 | 0.1306 |
| Population | 1 | 1.59 | 0.2156 | 1 | **4.36** | 0.0435 | 1 | 2.66 | 0.1110 | 1 | 0.11 | 0.7388 |
| Error | 38 | | | 38 | | | 38 | | | 38 | | |
| *Contrast variable: ambient – +4 °C* | | | | | | | | | | | | |
| Mean | 1 | **27.96** | <.0001 | 1 | **131.11** | <.0001 | 1 | **18.77** | 0.0001 | 1 | **26.39** | <.0001 |
| Population | 1 | 0.13 | 0.7193 | 1 | 0.44 | 0.5092 | 1 | 0.35 | 0.5568 | 1 | 0.51 | 0.4811 |
| Error | 38 | | | 38 | | | 38 | | | 38 | | |

**Table S2.** Simple-effect comparisons for SLWC and estimated LT in *I. pumila* genotypes from Dune and Woods populations analyzed across sequential temperature regimes (ambient, +1 °C and +4 °C). **A.** Within-population comparisons among temperature regimes were performed using two-sided paired *t*-tests (df = 19). **B.** Between-population comparisons within each temperature regime were performed using two-sided independent-samples *t*-tests (df = 38). No additional multiple-testing adjustment was applied to these follow-up comparisons. Mean diff. represents the mean difference between the two levels compared, calculated as the first level minus the second: for within-population comparisons, the first temperature regime minus the second; for between-population comparisons, Dune minus Woods. SE = standard error of the mean difference; *t*= *t*-statistic; *p* = *p*-value . Statistically significant *t*-values are shown in bold, while marginally significant results ($0.05 < p < 0.10$) are underlined. For trait acronyms and measurement units, see Table 1.

| **A.** | **SLWC** | | | | **LT** | | | |
|---|---|---|---|---|---|---|---|---|
| Source of variation | Mean diff. | SE | *t* | *p* | Mean diff. | SE | *t* | *p* |
| **Dune** | *Variable contrast: temperature regime* | | | | | | | |
| Ambient vs. +1 °C | -0.0030 | 0.0005 | **-5.38** | <.0001 | -0.0030 | 0.0006 | **-4.93** | <.0001 |
| Ambient - +4 °C | -0.0030 | 0.0004 | **-6.51** | <.0001 | -0.0020 | 0.0005 | **-4.78** | 0.0001 |
| +1 °C vs. +4 °C | 0.00006 | 0.0005 | 0.12 | 0.9044 | 0.0004 | 0.0006 | 0.75 | 0.4646 |
| **Woods** | *Variable contrast: temperature regime* | | | | | | | |
| Ambient vs. +1 °C | -0.0010 | 0.0003 | **-3.99** | 0.0008 | -0.0010 | 0.0007 | <u>-2.00</u> | 0.0604 |
| Ambient vs. +4 °C | -0.0030 | 0.0004 | **-6.78** | <.0001 | -0.0030 | 0.0007 | **-4.51** | 0.0002 |
| +1 °C vs. +4 °C | -0.0020 | 0.0004 | **-4.33** | 0.0004 | -0.0020 | 0.0004 | **-4.57** | 0.0002 |

| **B.** | **SLWC** | | | | **LT** | | | |
|---|---|---|---|---|---|---|---|---|
| Source of variation | Mean diff. | SE | *t* | *p* | Mean diff. | SE | *t* | *p* |
| | *Variable contrast: Dune - Woods* | | | | | | | |
| Ambient | -0.00043 | 0.0006 | -0.76 | 0.4520 | -0.00087 | 0.0007 | -1.32 | 0.1951 |
| +1 °C | 0.0013 | 0.0006 | **2.03** | 0.0498 | 0.0006 | 0.0008 | 0.83 | 0.4141 |
| +4 °C | -0.0006 | 0.0007 | -0.87 | 0.3879 | -0.0020 | 0.0007 | **-2.13** | 0.0396 |

**Table S3.** Coefficient of variation (CV%) for functional and mechanistic leaf traits in *I. pumila* genotypes originating from the Dune and Woods populations. A total of 40 genotypes (20 per population) were analyzed across sequential temperature regimes (ambient, +1 °C and +4 °C) in an environmentally controlled growth room. For trait acronyms and measurement units, see Table 1.

| TRAITS | DUNE | | | WOODS | | |
|---|---|---|---|---|---|---|
| | Ambient | +1 °C | +4 °C | Ambient | +1 °C | +4 °C |
| *FUNCTIONAL* | | | | | | |
| SLA | 4.9 | 6.5 | 9.2 | 8.2 | 9.0 | 10.6 |
| LDMC | 4.6 | 7.1 | 8.7 | 6.4 | 6.4 | 8.7 |
| *MECHANISTIC* | | | | | | |
| SLWC | 7.3 | 9.6 | 7.4 | 10.2 | 9.0 | 11.1 |
| LT | 6.2 | 8.2 | 6.5 | 9.6 | 9.0 | 9.3 |
| SD | 17.4 | 15.2 | 14.4 | 15.6 | 16.2 | 13.2 |
| $I_1$ | 10.9 | 15.4 | 13.4 | 18.4 | 19.1 | 17.8 |

**Table S4.** Comparisons of phenotypic variances in *I. pumila* genotypes within populations across temperature regimes and between populations within each temperature regime: **A.** within-population comparisons across temperature regimes using paired Pitman–Morgan tests (df = 18) and **B.** between-population comparisons within each temperature regime using two-sided *F*-tests for equality of variances ($df_1 = 19$, $df_2 = 19$). Here, *t* denotes the Pitman–Morgan test statistic, *F* the *F*-test statistic, and *p* the Holm-adjusted *p*-value. Statistically significant results ($p < 0.05$) are shown in bold, while marginally significant results ($0.05 < p < 0.10$) are underlined. For trait acronyms and measurement units, see Table 1.

| **A.** | Dune | | | | | | Woods | | | | | |
|---|---|---|---|---|---|---|---|---|---|---|---|---|
| | Ambient vs. +1 °C | | Ambient vs. +4 °C | | +1 °C vs. +4 °C | | Ambient vs. +1 °C | | Ambient vs. +4 °C | | +1 °C vs. +4 °C | |
| Traits | *t* | *p* | *t* | *p* | *t* | *p* | *t* | *p* | *t* | *p* | *t* | *p* |
| *Functional* | | | | | | | | | | | | |
| SLA | -1.288 | 0.214 | **-3.579** | 0.006 | -1.918 | 0.142 | -0.742 | 0.561 | -1.533 | 0.428 | -1.112 | 0.561 |
| LDMC | -1.555 | 0.275 | -2.235 | 0.115 | -0.806 | 0.414 | 0.361 | 1.000 | -0.564 | 1.000 | -0.896 | 1.000 |
| *Mechanistic* | | | | | | | | | | | | |
| SLWC | -1.869 | 0.234 | -0.725 | 0.503 | 1.185 | 0.503 | 0.468 | 0.646 | -1.288 | 0.428 | -1.708 | 0.314 |
| LT | -1.698 | 0.320 | -0.591 | 0.574 | 1.097 | 0.574 | 0.159 | 0.876 | -1.238 | 0.463 | -1.840 | 0.247 |
| SD | 0.416 | 0.683 | 1.695 | 0.322 | 1.034 | 0.630 | -0.155 | 0.878 | 1.172 | 0.513 | 1.593 | 0.386 |
| $I_1$ | -2.373 | 0.087 | -1.546 | 0.279 | 0.414 | 0.684 | -0.608 | 1.000 | -0.444 | 1.000 | 0.167 | 1.000 |

| **B.** | Dune vs. Woods | | | | | |
|---|---|---|---|---|---|---|
| | Ambient | | +1 °C | | +4 °C | |
| Traits | *F* | *p* | *F* | *p* | *F* | *p* |
| *Functional* | | | | | | |
| SLA | 0.433 | 0.226 | 0.600 | 0.548 | 0.955 | 0.921 |
| LDMC | 0.476 | 0.344 | 1.024 | 1.000 | 0.934 | 1.000 |
| *Mechanistic* | | | | | | |
| SLWC | 0.484 | 0.245 | 1.287 | 0.588 | 0.428 | 0.216 |
| LT | 0.398 | 0.103 | 0.910 | 0.840 | 0.328 | 0.057 |
| SD | 1.146 | 1.000 | 0.888 | 1.000 | 1.015 | 1.000 |
| $I_1$ | 0.485 | 0.317 | 0.898 | 1.000 | 0.812 | 1.000 |

**Table S5.** Spearman's correlation coefficients (ρ) and *p*-values for plasticity indices (*PI*v) of *I. pumila* genotypes originating from Dune and Woods populations across three temperature contrasts derived from the sequential temperature regimes: **A.** ambient vs. +1 °C, **B.** ambient vs. +4 °C, and **C.** +1 °C vs. +4 °C. In each panel, the lower triangular part presents correlation coefficients for genotypes from the Dune population, whereas the grey-shaded upper triangular part presents those for genotypes from the Woods population. Statistically significant correlations ($p < 0.05$) are shown in bold, while marginally significant correlations ($0.05 < p < 0.10$) are underlined. The reported *p*-values are unadjusted and interpreted descriptively. For trait acronyms and measurement units see Table 1.

| **A.**<br>**ambient vs. +1°C** | ***PI*v SLA** | ***PI*v LDMC** | ***PI*v SLWC** | ***PI*v LT** | ***PI*v SD** | ***PI*v $I_1$** |
|---|---|---|---|---|---|---|
| ***PI*v SLA** | | 0.322<br>0.166 | -0.293<br>0.210 | **-0.517**<br>0.020 | -0.296<br>0.205 | -0.060<br>0.801 |
| ***PI*v LDMC** | 0.032<br>0.895 | | 0.092<br>0.701 | -0.334<br>0.150 | <u>-0.441</u><br>0.052 | -0.047<br>0.845 |
| ***PI*v SLWC** | -0.051<br>0.831 | **0.463**<br>0.040 | | **0.465**<br>0.039 | 0.042<br>0.860 | -0.005<br>0.985 |
| ***PI*v LT** | -0.039<br>0.870 | 0.277<br>0.238 | **0.959**<br><.0001 | | 0.189<br>0.424 | -0.045<br>0.850 |
| ***PI*v SD** | -0.066<br>0.782 | -0.053<br>0.826 | -0.173<br>0.466 | -0.254<br>0.280 | | 0.195<br>0.409 |
| ***PI*v $I_1$** | 0.020<br>0.935 | <u>-0.400</u><br>0.081 | -0.014<br>0.955 | 0.098<br>0.682 | 0.116<br>0.627 | |

| **B.**<br>**ambient vs. +4°C** | ***PI*v SLA** | ***PI*v LDMC** | ***PI*v SLWC** | ***PI*v LT** | ***PI*v SD** | ***PI*v $I_1$** |
|---|---|---|---|---|---|---|
| ***PI*v SLA** | | **0.453**<br>**0.045** | -0.377<br>0.101 | -0.232<br>0.326 | 0.065<br>0.786 | 0.226<br>0.339 |
| ***PI*v LDMC** | 0.123<br>0.604 | | 0.147<br>0.535 | 0.000<br>1.000 | -0.257<br>0.274 | 0.134<br>0.574 |
| ***PI*v SLWC** | -0.009<br>0.970 | 0.289<br>0.217 | | **0.517**<br>**0.020** | -0.180<br>0.446 | -0.030<br>0.900 |
| ***PI*v LT** | 0.014<br>0.955 | 0.132<br>0.578 | **0.949**<br>**<.0001** | | <u>0.394</u><br>0.086 | -0.132<br>0.578 |
| ***PI*v SD** | -0.108<br>0.650 | 0.218<br>0.356 | 0.311<br>0.182 | 0.199<br>0.402 | | 0.012<br>0.960 |
| ***PI*v $I_1$** | 0.005<br>0.985 | -0.153<br>0.518 | -0.235<br>0.320 | -0.209<br>0.376 | -0.221<br>0.349 | |

| **C.**<br>**+1°C vs. +4°C** | ***PI*v SLA** | ***PI*v LDMC** | ***PI*v SLWC** | ***PI*v LT** | ***PI*v SD** | ***PI*v $I_1$** |
|---|---|---|---|---|---|---|
| ***PI*v SLA** | | 0.119<br>0.618 | -0.167<br>0.482 | -0.233<br>0.323 | -0.014<br>0.955 | 0.018<br>0.940 |
| ***PI*v LDMC** | 0.039<br>0.870 | | 0.362<br>0.116 | <u>0.402</u><br>0.079 | 0.147<br>0.537 | 0.377<br>0.101 |
| ***PI*v SLWC** | <u>0.408</u><br>0.074 | 0.017<br>0.945 | | **0.820**<br>**<.0001** | 0.100<br>0.675 | <u>0.432</u><br>0.057 |
| ***PI*v LT** | **0.662**<br>**0.002** | -0.023<br>0.925 | **0.844**<br>**<.0001** | | 0.026<br>0.912 | 0.326<br>0.160 |
| ***PI*v SD** | <u>-0.400</u><br>0.081 | 0.217<br>0.359 | -0.343<br>0.139 | <u>-0.391</u><br>0.088 | | 0.124<br>0.602 |
| ***PI*v $I_1$** | -0.066<br>0.782 | -0.098<br>0.682 | -0.084<br>0.724 | -0.099<br>0.677 | -0.063<br>0.791 | |

**Table S6**. Eigenvector coefficients for functional and mechanistic leaf traits for the first three principal components (PC1-PC3) in *I. pumila* genotypes originating from the Dune and Woods populations across sequential temperature regimes (ambient, +1 °C and +4 °C). LT represents estimated leaf thickness derived from SLA and LDMC following Vile et al. [32]. Eigenvector coefficients with absolute values greater than 0.400 are given in bold. Eigenvalues, the proportion of variance explained, cumulative proportion and the relative variance of eigenvalues (r*VE*), based on the full set of PCA eigenvalues, with 95% confidence intervals, are reported for each population and temperature regime.

| | | DUNE | | | WOODS | | |
|---|---|---|---|---|---|---|---|
| | Trait | PC1 | PC2 | PC3 | PC1 | PC2 | PC3 |
| | SLA | 0.275 | **0.624** | -0.359 | **-0.561** | -0.295 | -0.182 |
| | LDMC | **0.456** | -0.262 | 0.397 | -0.031 | **0.699** | 0.122 |
| | SLWC | **-0.577** | -0.033 | -0.105 | **0.564** | -0.107 | 0.143 |
| | LT | **-0.575** | -0.128 | -0.044 | **0.564** | -0.262 | 0.038 |
| | SD | 0.150 | **-0.679** | -0.169 | 0.146 | **0.575** | -0.384 |
| **Ambient** | $I_1$ | 0.174 | -0.249 | **-0.820** | 0.164 | -0.121 | **-0.885** |
| | Eigenvalue | 2.894 | 1.404 | 1.034 | 2.498 | 1.605 | 1.059 |
| | Proportion variance explained | 0.482 | 0.234 | 0.172 | 0.416 | 0.268 | 0.177 |
| | Cumulative proportion | | | 0.889 | | | 0.860 |
| | Relative coefficient of integration (95% Confidence interval) | | | | | | |
| | r*VE* | 0.191 (0.163-0.303) | | | 0.146 (0.135-0.304) | | |
| | | DUNE | | | WOODS | | |
| | Trait | PC1 | PC2 | PC3 | PC1 | PC2 | PC3 |
| | SLA | -0.262 | **-0.611** | -0.284 | **-0.512** | -0.166 | 0.275 |
| | LDMC | -0.357 | **0.444** | 0.330 | 0.175 | **0.687** | **-0.476** |
| | SLWC | **0.540** | 0.163 | -0.318 | **0.474** | **-0.444** | 0.013 |
| | LT | **0.536** | 0.243 | -0.241 | **0.520** | -0.297 | -0.004 |
| | SD | -0.239 | **0.544** | -0.063 | **0.426** | 0.202 | 0.175 |
| **+1 °C** | $I_1$ | **-0.409** | 0.218 | **-0.805** | 0.177 | **0.418** | **0.817** |
| | Eigenvalue | 2.962 | 1.833 | 0.579 | 3.118 | 1.403 | 0.896 |
| | Proportion variance explained | 0.494 | 0.306 | 0.096 | 0.520 | 0.234 | 0.149 |
| | Cumulative proportion | | | 0.896 | | | 0.903 |
| | Relative coefficient of integration (95% Confidence interval) | | | | | | |
| | r*VE* | 0.224 (0.188-0.373) | | | 0.224 (0.163-0.367) | | |
| | | DUNE | | | WOODS | | |
| | Trait | PC1 | PC2 | PC3 | PC1 | PC2 | PC3 |
| | SLA | 0.089 | **-0.747** | 0.042 | **0.531** | -0.222 | 0.025 |
| | LDMC | 0.344 | **0.615** | 0.111 | -0.183 | **0.645** | -0.386 |
| | SLWC | **-0.553** | 0.022 | -0.399 | **-0.493** | **-0.461** | -0.037 |
| | LT | **-0.550** | 0.212 | -0.315 | **-0.535** | -0.348 | -0.128 |
| | SD | 0.341 | -0.119 | **-0.632** | -0.367 | **0.417** | 0.106 |
| | $I_1$ | 0.385 | 0.064 | **-0.572** | -0.145 | 0.164 | **0.906** |
| **+4 °C** | Eigenvalue | 2.657 | 1.645 | 1.105 | 2.719 | 1.553 | 1.041 |
| | Proportion variance explained | 0.443 | 0.274 | 0.184 | 0.453 | 0.259 | 0.174 |
| | Cumulative proportion | | | 0.901 | | | 0.886 |
| | Relative coefficient of integration (95% Confidence interval) | | | | | | |
| | r*VE* | 0.174 (0.143-0.319) | | | 0.173 (0.134-0.351) | | |

**Table S7.** Results of nonparametric longitudinal analysis of plasticity indices (*PI*v) for functional and mechanistic leaf traits of *I. pumila* genotypes originating from Dune and Woods populations across three temperature contrasts (ambient vs. +1 °C, ambient vs. +4 °C and +1 °C vs. +4 °C). ANOVA-type statistics (ATS), degrees of freedom (df) and *p*-values are presented for the effects of population, temperature contrast and their interaction. Statistically significant ATS values ($p < 0.05$) are shown in bold and marginally significant ($0.05 < p < 0.10$) are underlined.

| Trait | Population (P) | | Temperature (T) | | P x T | |
|---|---|---|---|---|---|---|
| | ATS | *p* | ATS | *p* | ATS | *p* |
| SLA | 2.34 (1) | 0.126 | **4.61 (1.91)** | 0.011 | 0.52 (1.91) | 0.585 |
| LDMC | 0.03 (1) | 0.862 | **37.89 (1.40)** | <0.001 | **9.09 (1.40)** | <0.001 |
| SLWC | 1.77 (1) | 0.183 | **9.48 (1.91)** | <0.001 | **6.40 (1.91)** | 0.002 |
| LT | 0.96 (1) | 0.327 | **7.53 (1.85)** | <0.001 | **4.16 (1.85)** | 0.018 |
| SD | 2.06 (1) | 0.151 | 0.15 (1.99) | 0.857 | 1.16 (1.99) | 0.315 |
| I1 | <u>2.94 (1)</u> | 0.086 | 2.10 (1.96) | 0.123 | 0.04 (1.96) | 0.956 |

**Table S8.** Sensitivity analyses of trait covariance and phenotypic integration after exclusion of SLWC and estimated LT, using the reduced four-trait set comprising SLA, LDMC, SD and $I_1$.

**A.** Principal component analysis (PCA) and relative variance of eigenvalues (r*VE*) across temperature regimes in Dune and Woods genotypes. Eigenvector coefficients with absolute values > 0.400 are shown in bold; eigenvalues, proportion of variance explained, cumulative proportion and r*VE* with 95% confidence intervals are also reported.

**B.** Exact Mantel comparisons of four-trait correlation matrices among temperature regimes within populations and between populations within each temperature regime; *p*-values were adjusted across the nine comparisons using the Benjamini–Hochberg (BH) procedure.

**C.** Pearson correlations of the reduced mechanistic composite (SD and $I_1$) across temperature regimes within each population.

**D.** Pearson correlations between the functional composite (SLA and LDMC) and the reduced mechanistic composite (SD and $I_1$) within each population and temperature regime. Pearson p-values in panels C and D are unadjusted and interpreted descriptively. Statistically significant Pearson correlations ($p < 0.05$) are shown in bold, while marginally significant correlations ($0.05 < p < 0.10$) are underlined.

| **A.** | Trait | **DUNE** | | | **WOODS** | | |
|---|---|---|---|---|---|---|---|
| | | PC1 | PC2 | PC3 | PC1 | PC2 | PC3 |
| **Ambient** | SLA | 0.352 | **-0.744** | 0.254 | 0.030 | -0.352 | **0.920** |
| | LDMC | **-0.478** | -0.055 | **0.858** | **-0.693** | -0.298 | -0.207 |
| | SD | **-0.693** | 0.053 | -0.241 | **-0.717** | 0.196 | 0.216 |
| | $I_1$ | **-0.410** | **-0.664** | -0.375 | -0.064 | **0.865** | 0.255 |
| | Eigenvalue | 1.462 | 1.130 | 0.876 | 1.361 | 1.113 | 0.999 |
| | Proportion variance explained | 0.366 | 0.282 | 0.219 | 0.340 | 0.278 | 0.250 |
| | Cumulative proportion | | | 0.867 | | | 0.868 |
| | Relative coefficient of integration (95% Confidence interval) | | | | | | |
| | r*VE* | 0.039 (0.026-0.194) | | | 0.031 (0.019-0.204) | | |
| | Trait | **DUNE** | | | **WOODS** | | |
| | | PC1 | PC2 | PC3 | PC1 | PC2 | PC3 |
| **+1 °C** | SLA | -0.267 | **0.812** | -0.112 | **-0.495** | **0.511** | **0.520** |
| | LDMC | **0.589** | 0.042 | **0.702** | **0.491** | -0.384 | **0.778** |
| | SD | **0.581** | -0.148 | **-0.699** | **0.564** | 0.202 | -0.326 |
| | $I_1$ | **0.495** | **0.562** | -0.076 | **0.442** | **0.741** | 0.134 |
| | Eigenvalue | 2.027 | 1.169 | 0.500 | 1.787 | 0.904 | 0.714 |
| | Proportion variance explained | 0.507 | 0.292 | 0.125 | 0.447 | 0.226 | 0.178 |
| | Cumulative proportion | | | 0.924 | | | 0.851 |
| | Relative coefficient of integration (95% Confidence interval) | | | | | | |
| | r*VE* | 0.152 (0.072-0.326) | | | 0.073 (0.035-0.219) | | |
| | Trait | **DUNE** | | | **WOODS** | | |
| | | PC1 | PC2 | PC3 | PC1 | PC2 | PC3 |
| **+4 °C** | SLA | **0.441** | **-0.588** | 0.323 | **-0.616** | 0.261 | 0.303 |
| | LDMC | **-0.648** | 0.292 | 0.144 | **0.650** | 0.003 | -0.272 |
| | SD | -0.355 | **-0.593** | **-0.720** | **0.435** | **0.538** | **0.710** |
| | $I_1$ | **-0.510** | **-0.467** | **0.598** | -0.094 | **0.802** | **-0.574** |
| | Eigenvalue | 1.754 | 1.480 | 0.497 | 1.902 | 1.241 | 0.546 |
| | Proportion variance explained | 0.439 | 0.370 | 0.124 | 0.475 | 0.310 | 0.136 |
| | Cumulative proportion | | | 0.933 | | | 0.922 |
| | Relative coefficient of integration (95% Confidence interval) | | | | | | |
| | r*VE* | 0.132 (0.094-0.302) | | | 0.129 (0.090-0.264) | | |

**B.**

| **Comparison type** | **Temperature comparison** | Mantel r | *p* | BH-adjusted *p* |
|---|---|---|---|---|
| Dune | Ambient vs. +1 °C | 0.888 | 0.042 | 0.125 |
| | Ambient vs. +4 °C | 0.421 | 0.292 | 0.292 |
| | +1 °C vs. +4 °C | 0.644 | 0.208 | 0.234 |
| Woods | Ambient vs. +1 °C | 0.514 | 0.167 | 0.234 |
| | Ambient vs. +4 °C | 0.557 | 0.042 | 0.125 |
| | +1 °C vs. +4 °C | 0.734 | 0.042 | 0.125 |
| Between populations | Ambient | 0.431 | 0.208 | 0.234 |
| | +1 °C | 0.942 | 0.083 | 0.188 |
| | +4 °C | 0.702 | 0.125 | 0.225 |

**C.**

| **Population** | **Temperature comparison** | Pearson r | *p* |
|---|---|---|---|
| Dune | Ambient vs. +1 °C | **0.510** | 0.021 |
| | Ambient vs. +4 °C | **0.532** | 0.016 |
| | +1 °C vs. +4 °C | **0.467** | 0.038 |
| Woods | Ambient vs. +1 °C | **0.498** | 0.026 |
| | Ambient vs. +4 °C | **0.504** | 0.023 |
| | +1 °C vs. +4 °C | **0.581** | 0.007 |

**D.**

| **Population** | **Temperature regime** | Pearson r | *p* |
|---|---|---|---|
| Dune | Ambient | 0.098 | 0.680 |
| | +1 °C | <u>0.379</u> | 0.099 |
| | +4 °C | <u>0.396</u> | 0.084 |
| Woods | Ambient | 0.085 | 0.722 |
| | +1 °C | -0.075 | 0.754 |
| | +4 °C | 0.270 | 0.250 |

**Table S9.** Sensitivity analysis of Pearson correlations involving functional and mechanistic composite trait groups in *I. pumila* genotypes from Dune and Woods populations. Pearson correlation coefficients (r) and *p*-values obtained using min–max normalization separately within each temperature regime (regime-specific) are compared with those obtained using common minimum and maximum values calculated for each trait across both populations and all three temperature regimes (common). Statistically significant correlations ($p < 0.05$) are shown in bold. Pearson *p*-values are unadjusted and interpreted descriptively. Marginally significant correlations ($0.05 < p < 0.10$) are underlined. The pattern of statistical significance remained unchanged across all 30 comparisons.

**A.** Cross-temperature correlations within the functional composite (SLA and LDMC), identical for the six-trait and four-trait sets.

**B.** Cross-temperature correlations within the mechanistic composite in the complete six-trait set (SLWC, estimated LT, SD and $I_1$).

**C.** Cross-temperature correlations within the mechanistic composite in the reduced four-trait set (SD and $I_1$).

**D.** Correlations between the functional and mechanistic composites within each temperature regime in the complete six-trait set.

**E.** Correlations between the functional and mechanistic composites within each temperature regime in the reduced four-trait set.

| **A.** Population | Temperature comparison | Regime-specific | | Common | |
|---|---|---|---|---|---|
| | | **Pearson r** | ***p*** | **Pearson r** | ***p*** |
| Dune | Ambient vs. +1 °C | 0.310 | 0.183 | 0.324 | 0.163 |
| | Ambient vs. +4 °C | 0.274 | 0.243 | 0.252 | 0.284 |
| | +1 °C vs. +4 °C | 0.396 | 0.084 | 0.261 | 0.267 |
| Woods | Ambient vs. +1 °C | 0.404 | 0.077 | 0.430 | 0.058 |
| | Ambient vs. +4 °C | 0.049 | 0.838 | 0.127 | 0.592 |
| | +1 °C vs. +4 °C | **0.586** | 0.007 | **0.623** | 0.003 |

| **B.** Population | Temperature comparison | Regime-specific | | Common | |
|---|---|---|---|---|---|
| | | **Pearson r** | ***p*** | **Pearson r** | ***p*** |
| Dune | Ambient vs. +1 °C | **0.454** | 0.045 | **0.482** | 0.031 |
| | Ambient vs. +4 °C | **0.574** | 0.008 | **0.503** | 0.024 |
| | +1 °C vs. +4 °C | **0.611** | 0.004 | **0.520** | 0.019 |
| Woods | Ambient vs. +1 °C | **0.624** | 0.003 | **0.650** | 0.002 |
| | Ambient vs. +4 °C | **0.691** | 0.0007 | **0.724** | 0.0003 |
| | +1 °C vs. +4 °C | **0.753** | 0.0001 | **0.816** | <0.0001 |

| C. Population | Temperature comparison | Regime-specific | | Common | |
|---|---|---|---|---|---|
| | | Pearson r | *p* | Pearson r | *p* |
| Dune | Ambient vs. +1 °C | **0.510** | 0.021 | **0.452** | 0.046 |
| | Ambient vs. +4 °C | **0.532** | 0.016 | **0.489** | 0.029 |
| | +1 °C vs. +4 °C | **0.467** | 0.038 | **0.464** | 0.039 |
| Woods | Ambient vs. +1 °C | **0.498** | 0.026 | **0.479** | 0.033 |
| | Ambient vs. +4 °C | **0.504** | 0.023 | **0.536** | 0.015 |
| | +1 °C vs. +4 °C | **0.581** | 0.007 | **0.590** | 0.006 |

| D. Population | Temperature regime | Regime-specific | | Common | |
|---|---|---|---|---|---|
| | | Pearson r | *p* | Pearson r | *p* |
| Dune | Ambient | **-0.639** | 0.002 | **-0.679** | 0.001 |
| | +1 °C | **-0.588** | 0.006 | **-0.518** | 0.019 |
| | +4 °C | -0.284 | 0.224 | -0.401 | 0.080 |
| Woods | Ambient | **-0.478** | 0.033 | **-0.520** | 0.019 |
| | +1 °C | **-0.665** | 0.001 | **-0.616** | 0.004 |
| | +4 °C | **-0.490** | 0.029 | **-0.612** | 0.004 |

| E. Population | Temperature regime | Regime-specific | | Common | |
|---|---|---|---|---|---|
| | | Pearson r | *p* | Pearson r | *p* |
| Dune | Ambient | 0.098 | 0.680 | 0.044 | 0.854 |
| | +1 °C | 0.379 | 0.099 | 0.376 | 0.102 |
| | +4 °C | 0.396 | 0.084 | 0.383 | 0.095 |
| Woods | Ambient | 0.085 | 0.722 | 0.128 | 0.591 |
| | +1 °C | -0.075 | 0.754 | -0.060 | 0.803 |
| | +4 °C | 0.270 | 0.250 | 0.230 | 0.329 |
